# Gene pool transmission of multidrug resistance among *Campylobacter* from livestock, sewage and human disease

**DOI:** 10.1111/1462-2920.14760

**Published:** 2019-08-27

**Authors:** Evangelos Mourkas, Diego Florez‐Cuadrado, Ben Pascoe, Jessica K. Calland, Sion C. Bayliss, Leonardos Mageiros, Guillaume Méric, Matthew D. Hitchings, Alberto Quesada, Concepción Porrero, María Ugarte‐Ruiz, José Gutiérrez‐Fernández, Lucas Domínguez, Samuel K. Sheppard

**Affiliations:** ^1^ The Milner Centre for Evolution, Department of Biology and Biochemistry University of Bath BA27AY Bath UK; ^2^ VISAVET Health Surveillance Centre Universidad Complutense Madrid Madrid Spain; ^3^ MRC CLIMB Consortium University of Bath Bath UK; ^4^ Cambridge Baker Systems Genomics Initiative Baker Heart and Diabetes Institute, 75 Commercial Rd Melbourne 3004 Victoria Australia; ^5^ Department of Infectious Diseases, Central Clinical School Monash University Melbourne Victoria 3004 Australia; ^6^ Swansea University Medical School Swansea Swansea UK; ^7^ Department of Biochemistry, Molecular Biology and Genetics, Faculty of Veterinary Medicine University of Extremadura Cáceres Spain; ^8^ Department of Microbiology University of Granada Granada Spain; ^9^ Department of Animal Health, Faculty of Veterinary Medicine Universidad Complutense Madrid Madrid Spain; ^10^ Department of Zoology University of Oxford Oxford UK

## Abstract

The use of antimicrobials in human and veterinary medicine has coincided with a rise in antimicrobial resistance (AMR) in the food‐borne pathogens *Campylobacter jejuni* and *Campylobacter coli*. Faecal contamination from the main reservoir hosts (livestock, especially poultry) is the principal route of human infection but little is known about the spread of AMR among source and sink populations. In particular, questions remain about how *Campylobacter* resistomes interact between species and hosts, and the potential role of sewage as a conduit for the spread of AMR. Here, we investigate the genomic variation associated with AMR in 168 *C. jejuni* and 92 *C. coli* strains isolated from humans, livestock and urban effluents in Spain. AMR was tested in vitro and isolate genomes were sequenced and screened for putative AMR genes and alleles. Genes associated with resistance to multiple drug classes were observed in both species and were commonly present in multidrug‐resistant genomic islands (GIs), often located on plasmids or mobile elements. In many cases, these loci had alleles that were shared among *C. jejuni* and *C. coli* consistent with horizontal transfer. Our results suggest that specific antibiotic resistance genes have spread among *Campylobacter* isolated from humans, animals and the environment.

## Introduction

*Campylobacter* is the leading cause of bacterial gastroenteritis in the European Union (EU) (Food and Authority, 2019). The most common pathogenic species, *C. jejuni* and *C. coli*, were responsible for over 245,658 cases of campylobacteriosis in the EU in 2016, surpassing disease caused by *Escherichia coli*, *Salmonella* and *Listeria* (Food and Authority, 2019). *Campylobacter* are a common constituent of the gut microbiota of livestock including poultry, ruminants and pigs (Sheppard *et al*., 2009a, 2011; Sproston *et al*., 2011), and are also found in wild birds (Sheppard *et al*., 2010a, 2010b; Griekspoor *et al*., 2013; Cody *et al*., 2015; Atterby *et al*., 2018) and environmental sources (Dingle *et al*., 2001; Colles *et al*., 2003; Sheppard *et al*., 2009a). Human infection is typically associated with the consumption of contaminated meat (Fravalo *et al*., 2009; Hermans *et al*., 2012; Guyard‐Nicodème *et al*., 2013) and causes acute gastroenteritis and is self‐limiting after 3–5 days. In severe cases, antibiotic treatment may be required with fluoroquinolones and macrolides being the drugs of choice (Acheson and Allos, 2001).

Despite the ban on the use of antibiotics as growth promoters in animal feed in 2006 in the EU (Castanon, 2007), antimicrobial resistance (AMR) is still common among bacteria of the gastrointestinal tract of farmed animals (Sheppard *et al*., 2009a,b; Sproston *et al*., 2011). According to the latest European Centre for Disease Prevention and Control (ECDC) report in 2017, *C. jejuni* and *C. coli* isolates of clinical and animal origin showed high levels of resistance to both ciprofloxacin and tetracycline (Food and Authority, 2019). Furthermore, *C. coli* from clinical and animal samples have displayed resistance to macrolides including erythromycin and the aminoglycoside streptomycin (Food and Authority, 2019). More worryingly, there is an apparent trend towards multidrug resistance (MDR), particularly among *C. coli* that regularly harbour different AMR genes simultaneously within the genome of a single isolate (Luangtongkum *et al*., 2009; Pascoe *et al*., 2017; Food and Authority, 2019).

Mechanisms of resistance are well documented for several drug classes including fluoroquinolones, tetracyclines, macrolides, aminoglycosides and β‐lactams. Fluoroquinolone treatment was traditionally the first line of defence against campylobacteriosis but resistance has rapidly increased among strains (Sproston *et al*., 2018), potentially because it requires only a single point mutation in the genome (in the *gyrA* gene; Luo *et al*., 2003; Gibreel, 2006; Payot *et al*., 2006; Luangtongkum *et al*., 2009). This has led to a shift in treatment in favour of erythromycin prescription (Nachamkin *et al*., 2000; Gibreel, 2006), where resistance arises from specific point mutations in 23S rRNA and develops relatively slowly (Lapierre *et al*., 2016). However, in 2014, erythromycin resistance was found in animal and clinical isolates that carried an rRNA methylating enzyme, the *ermB* gene (Qin *et al*., 2014; Wang *et al*., 2014). Two years later the *ermB* gene was detected in *C. coli* isolates from turkeys and chickens in Spain suggesting the mobilization of this gene through horizontal gene transfer (HGT; Florez‐Cuadrado *et al*., 2016, 2018). Tetracycline resistance, associated with the *tetO* gene encoding a ribosomal protection protein, has also been observed in *Campylobacter* since 1987 (Sougakoff *et al*., 1987) and new enzymes conferring resistance to aminoglycosides continue to be discovered in *Campylobacter* (Lambert *et al*., 1985; Iovine, 2013; Zhao *et al*., 2016). In addition to these emerging trends, *Campylobacter* is known to have ‘natural’ resistance to β‐lactams, such as penicillin, in large part due to the ubiquity of the *bla*_OXA‐61_ gene (Alfredson and Korolik, 2005; Griggs *et al*., 2009). As a result of the widespread resistance to multiple antibiotic classes, it is no surprise that *Campylobacter* is a high priority pathogen on the recently published World Health Organization (WHO) list of bacteria, for which new antibiotics are urgently needed (WHO, 2017).

Many studies have highlighted the potential for transmission of AMR bacteria between agricultural animals and humans following extended use of antibiotics (Boerlin and Reid‐Smith, 2008; Huttner *et al*., 2013). However, controversy surrounding evidence for a direct link is confounded by inconsistencies in interpreting what constitutes the spread of resistance. Broadly, the spread of AMR can be defined as a clonal transmission or gene pool transmission. In clonal transmission, bacteria that have acquired AMR in one niche are transmitted to another where they retain resistance, such as in the survival of resistant *Campylobacter* through the food production chain to infect humans (Yahara *et al*., 2017). In gene pool transmission, HGT facilitates the spread of resistance genes between strains and species and the movement of genes (rather than clones) into multiple genetic backgrounds can be seen to spread AMR. Efforts to reduce AMR and conserve the remaining efficacy of existing drugs are focussed on the judicious use of antibiotics in animals and humans. In this context, it is advantageous to consider gene pool transmission as this is directly influenced by the selection pressure to maintain resistance in a given environment.

*Campylobacter jejuni* and *C. coli* can evolve rapidly, accumulating large numbers of nucleotide substitutions through mutation and recombination (Wilson *et al*., 2009; Sheppard *et al*., 2010a, 2010b; Dearlove *et al*., 2016). This can lead to *de novo* development of AMR through point mutation as well as the acquisition of resistance elements from other bacteria through HGT (Yahara *et al*., 2014, 2016). HGT has a major role in the mobilization of AMR not only within bacterial species but even across species boundaries. For example, the *tetO* gene that confers resistance to tetracycline in *Campylobacter* (Taylor *et al*., 1983; Batchelor, 2004) is believed to have originated via HGT from a Gram‐positive bacterium, potentially mediated by plasmid transfer (Taylor *et al*., 1983; Taylor, 1986; Batchelor, 2004). Interspecies genetic exchange requires some degree of niche overlap or physical proximity of strains. However, while there is some understanding of host niche segregation and clonal transmission of particular *Campylobacter* lineages (Sheppard *et al*., 2009a, 2010a, 2010b, 2014), there is limited quantitative information about the transmission dynamics of AMR genes between human, animal and environmental gene pools (gene pool transmission) in this genus.

In this study, we sequence the genome of isolates from a survey of AMR *Campylobacter* from multiple sources in Spain. Multidrug resistance phenotypes are quantified *in vitro* and compared to putative genomic determinants identified from over 2,000 known AMR genes. The co‐localization of these genes within resistance islands is examined and the allelic variation is compared among isolates from different sample sources. These analyses provide a basis for considering the interaction of different AMR gene pools and the potential source/sink contribution of livestock, humans and sewage effluents to the *Campylobacter* resistome.

## Results

### *Enhanced* in vitro *MDR in* C. coli *compared to* C. jejuni

We collected 168 *C. jejuni* and 92 *C. coli* isolates of human, animal and sewage origin (Supporting Information Table S1). *In vitro* resistance to six antibiotics (ciprofloxacin, nalidixic acid, tetracycline, erythromycin, streptomycin and gentamicin) of isolates of animal origin (Table 1, Supporting Information Table S2) was compared to resistance profiles of isolates of human and sewage origin (Table 1, Supporting Information Table S2). All *Campylobacter* isolates that were resistant to both ciprofloxacin and nalidixic acid were referred to as ciprofloxacin resistant only because resistance is conferred by SNPs in the same gene. The highest proportion of AMR was to ciprofloxacin (146/163; 90.1% for *C. jejuni* and 86/91; 94.5% for *C. coli*) and tetracycline (149/163; 91.4% for *C. jejuni* and 86/91; 94.5% for *C. coli*), followed by streptomycin (24/163; 14.7% for *C. jejuni* and 58/91; 63.7% for *C. coli*), erythromycin (4/162; 2.5% for *C. jejuni* and 23/91; 25.3% for *C. coli*) and gentamicin (2/163; 1.2% for *C. jejuni* and 10/91; 11% for *C. coli*; Table 1, Supporting Information Table S2). Higher prevalence of resistance was observed in *C. coli* isolates to erythromycin, streptomycin and gentamicin compared to *C. jejuni* (Fisher's exact test; *p* < 0.001). Typically, an isolate is considered multidrug resistant when it is resistant to at least three different classes of antibiotics (European Centre for Disease Prevention and Control [ECDC] & European Food Safety Authority [EFSA], 2015). Based on this criterion, more *C. coli* isolates were MDR (49/91; 53.8%) than *C. jejuni* (27/163; 16.6%; Table 2). All *C. coli* isolates were resistant to at least one antibiotic. (Table 2). Six (out of 163; 3.7%) *C. jejuni* isolates were sensitive to all tested antibiotics. Most of the isolates tested were resistant to both ciprofloxacin and tetracycline (140/163 or 85.9% *C. jejuni* and 82/91 or 90.1% *C. coli*), of which 52 *C. coli* isolates (57.1%) were also resistant to streptomycin compared to 24 *C. jejuni* isolates (14.7%) and nine *C. coli* isolates (9.9%) were also resistant to gentamicin compared to two *C. jejuni* isolates (1.23%; Table 2).

**Table 1:** Drug resistance profiles of 254 *Campylobacter* isolates from humans, animals and sewage tested in the lab.

	*Campylobacter jejuni*				*Campylobacter coli*			
Antibiotics^a^	Animals	Humans	Sewage	Total	Animals	Humans	Sewage	Total
Ciprofloxacin	36/44 (81.8%)	106/115 (88.7%)	4/4 (100%)	146/163 (90.12%)	11/11 (100%)	32/33 (97%)	43/47 (91.5%)	86/91 (94.5%)
Nalidixic acid	35/44 (79.54%)	78/115 (67.83%)	3/4 (75%)	116/163 (71.16%)	11/11 (100%)	30/33 (90.1%)	43/47 (91.5%)	84/91 (92.31%)
Tetracycline	39/44 (88.6%)	108/115 (93.91%)	2/4 (50%)	149/163 (91.41%)	11/11 (100%)	31/33 (94%)	44/47 (93.6%)	86/91 (94.5%)
Erythromycin	3/44 (6.8%)	1/115 (0.87%)	0/4 (0%)	4/163 (2.45%)	10/11 (90.1%)	6/33 (18.2%)	7/47 (14.9%)	23/91 (25.3%)
Streptomycin	15/44 (34.1%)	9/115 (7.83%)	0/4 (0%)	24/163 (14.72%)	10/11 (90.1%)	18/33 (54.5%)	30/47 (63.8%)	58/91 (63.7%)
Gentamicin	0/44 (0%)	2/115 (1.7%)	0/4 (0%)	2/163 (1.23%)	4/11 (36.4%)	2/33 (6.1%)	4/47 (8.51%)	10/91 (11%)
Total number of isolates	44	115	4	163	11	33	47	91

a Antibiotics resistance to: C, ciprofloxacin; T, tetracycline; E, erythromycin; S, streptomycin; G, gentamicin.

**Table 2:** Multidrug resistant (in bold) and non‐multidrug resistant *Campylobacter* isolates (*n* = 254) from humans, animals and sewage.

		*Campylobacter jejuni* (*n* = 162)			*Campylobacter coli* (*n* = 91)		
	Antibiotics^a^	Animals	Humans	Sewage	Animals	Humans	Sewage
	**CTESG**	–	–	–	4/11 (36.4%)	1/33 (3%)	–
	**CTES**	–	–	–	5/11 (45.5%)	4/33 (12.1%)	5/47 (10.6)
Multiresistant	**CTSG**	–	2/115 (1.7%)	–	–	1/33 (3%)	3/47 (6.4%)
	**CTS**	15/44 (34.1%)	7/115 (6.9%)	–	1/11 (9.1%)	11/33 (33.3%)	17/47 (36.2%)
	**CTE**	2/44 (4.5%)	1/115 (0/9%)	–	1/11 (9.1%)	1/33 (3%)	2/47 (4.3%)
	CT	16/44 (36.4%)	95/115 (82.6%)	2/4 (50%)	–	12/33 (36.4%)	13/47 (27.7%)
	CS	–	–	–	–	–	1/47 (2.1%)
	TE	1/44 (2.27%)	–	–	–	–	–
Non‐multiresistant	TS	1/44 (2.27%)	–	–	–	1/33 (3%)	4/47 (8.5%)
	C	3/44 (6.8%)	1/115 (0.9%)	1/4 (25%)	–	2/33 (6.1%)	2/47 (4.25%)
	T	4/44 (11.4%)	5/115 (4.4%)	–	–	–	–
Non‐resistant	Sensitive	2/44 (4.5%)	4/115 (3.5%)	–	–	–	–
Total number of non‐multidrug resistant		27/44 (61.36%)	101/115 (8.69%)	4/4 (100%)	–	15/33 (45.45%)	27/47 (57.44%)
Total number of multidrug resistant		17/44 (38.63%)	10/115 (87.82%)	–	11/11 (100%)	18/33 (54.54%)	20/47 (42.55%)
Total number of isolates		44	115	4	11	33	47

a Antibiotics resistance to: C, ciprofloxacin; T, tetracycline; E, erythromycin; S, streptomycin; G, gentamicin.

### *AMR isolates are distributed across highly structured populations*

**Figure 1:**
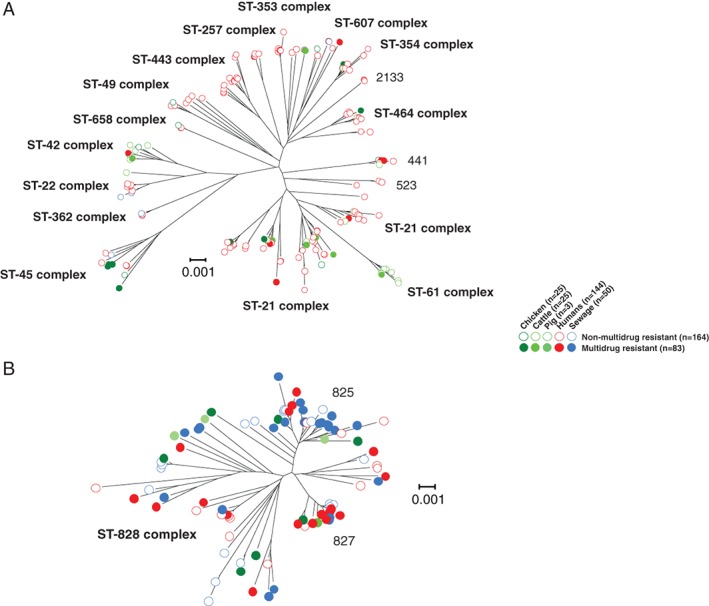
Phylogeny of antimicrobial resistant *Campylobacter*. Trees were reconstructed for 167 *C. jejuni* (A) and 92 *C. coli* (B) using concatenated gene‐by‐gene alignments of 595 core genes using the neighbour‐joining algorithm. Common sequence types and clonal complexes, defined by MLST, are indicated on the trees. Multidrug resistant isolates from chickens (dark green), cattle (intermediate green), pigs (light green), humans (red) and sewage (blue) are indicated with a filled circle, while the non‐multidrug resistant isolates are indicated with an open circle. The scale bars represent the number of substitutions per site. [Color figure can be viewed at http://wileyonlinelibrary.com]

High levels of AMR observed in laboratory assays could indicate either an abundance of low diversity AMR clones or proliferation of AMR in multiple lineages. To investigate this, we analysed the population genomic structure of AMR isolates. The core genome phylogeny revealed that AMR isolates belonged to genome sequence clusters consistent with existing multilocus sequence typing (MLST) Sequence Type (ST) and clonal complex designations (Dingle *et al*., 2001; Miller, 2006; Fig. 1). *Campylobacter jejuni* isolates of chicken and cattle origin were mainly of host generalist (ST‐21, ST‐48, ST‐206 and ST‐45) clonal complexes (Sheppard *et al*., 2010a, 2010b, 2014; Fig. 1A, Supporting Information Table S1). Cattle isolates also belonged to ST‐61 and ST‐42 cattle associated clonal complexes, while human clinical isolates contained isolates of these generalist and cattle associated clonal complexes as well as additional generalist clonal complexes (ST‐22, ST‐52) and chicken associated clonal complexes (ST‐257, ST‐353, ST‐354, ST‐443, ST464, ST‐574 and ST‐658; Fig. 1A, Supporting Information Table S1). *Campylobacter jejuni* isolates from sewage belonged to ST‐362, a human associated complex and generalist ST‐22, ST‐45 and ST‐607 complexes (Fig. 1A, Supporting Information Table S1). Multidrug resistant *C. jejuni* isolates (27/167) were from generalist (ST‐21, ST‐206, ST‐45, ST‐52) complexes, chicken associated complexes (ST‐354, ST‐460 and ST‐464) and cattle associated complexes (ST‐42 and ST‐61; Fig. 1A, Supporting Information Table S1). *Campylobacter coli* isolates represented 28 different STs, all of which belonged to the ST‐828 clonal complex. The most abundant STs were 825 and 827, constituting 20.7% and 17.4% of all *C. coli* isolates (Fig. 1B, Supporting Information Table S1). The proportion of *C. coli* isolates displaying MDR (60.9%) was considerably higher than within *C. jejuni* (16.1%), nearly half of which were isolated from sewage highlighting the potential importance of urban effluents as reservoirs of AMR genes (Fig. 1B, Table 2). Clearly, diversity within this complex is lower than in agricultural/clinical *C. jejuni* and one might consider ST‐828 complex to be a single clone. However, as illustrated (Fig. 1B), AMR is found in divergent lineages within the ST‐828 complex and, importantly, is also absent in some closely related strains. This pattern is inconsistent with the proliferation of a clone that acquired AMR genes in a single ancestral acquisition event. Rather it suggests horizontal transfer of AMR genes among sublineages.

### Campylobacter coli *genomes harbour more AMR genes than* C. jejuni

**Figure 2:**
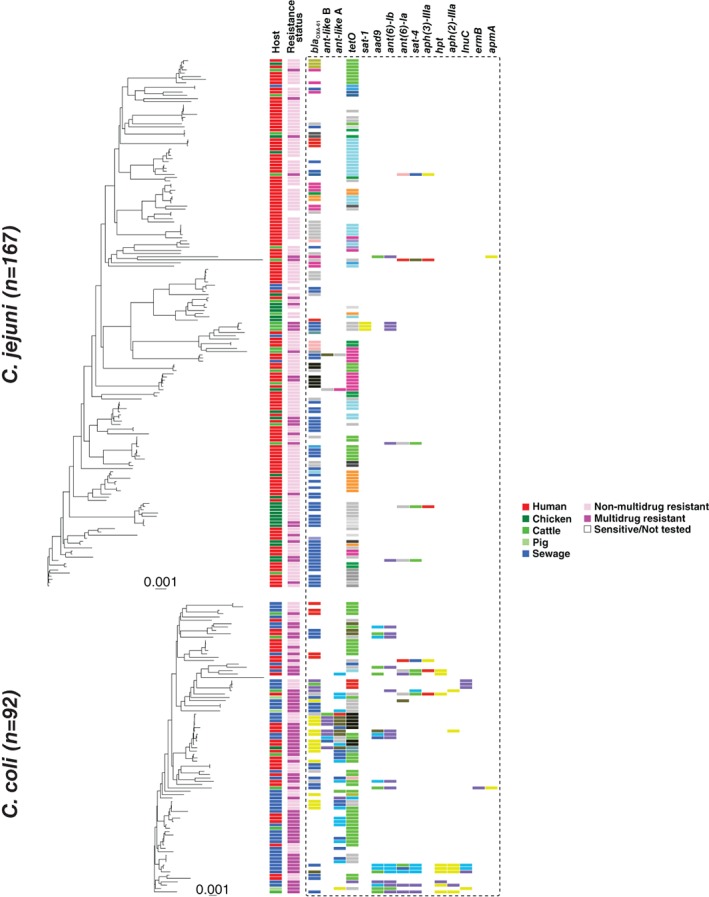
Presence and allelic diversity of 15 antimicrobial resistance genes in *C. jejuni* and *C. coli* genomes. Phylogenetic trees were reconstructed using gene‐by‐gene concatenated alignments of 595 core genes, and the neighbour‐joining algorithm for for 167 *C. jejuni* (A) and 92 *C. coli* (B). Isolate source is shown in the first column for chicken (dark green), cattle (green), pigs (light green), humans (red) and sewage (blue). The second column indicates the resistance status of each isolate as multidrug resistant (dark pink), non‐multidrug resistance (light pink) or not tested (white). Remaining columns indicate allelic variation at known resistance gene loci, with identical alleles coloured with the same colour. The scale represents the number of substitutions per site. [Color figure can be viewed at http://wileyonlinelibrary.com]

The genome sequences of all *Campylobacter* isolates were compared to 2,158, 2,280 and 4,324 known antibiotic resistance genes and alleles from the Comprehensive Antibiotic Resistance Database (CARD; Cameron and Gaynor, 2014), ResFinder (Zankari *et al*., 2012) and the National Center for Biothechnology Information (NCBI) databases respectively. The analysis revealed the presence of 18 AMR genes including: *cmeA*, *cmeB*, *cmeC*, bla_OXA‐61_, *tetO*, *ant‐like* A, *ant‐like* B, *ant(6)‐Ia*, *sat‐1*, *sat‐4*, *lnuC*, *ant(6)‐Ib*, *aad9*, *aph(3)‐IIIa*, *aph(2)‐IIIa*, *hpt*, *apmA* and *ermB* (Fig. 2, Table 3; Trieu‐Cuot *et al*., 1985; Sougakoff *et al*., 1987; Achard *et al*., 2005; Alfredson and Korolik, 2005; Griggs *et al*., 2009; Qin *et al*., 2012; Toth *et al*., 2013; Cameron and Gaynor, 2014; Zhao *et al*., 2016; Florez‐Cuadrado *et al*., 2016; Olkkola *et al*., 2016; Yao *et al*., 2017). The *cmeA*, *cmeB* and *cmeC* genes, associated with efflux pump function, were present in all isolates. The *bla*_OXA‐61_ and *tetO* genes were common in resistant *C. jejuni* and *C. coli* isolates (Fig. 2, Table 3). The genes *ant‐like* A and *ant‐like* B have been described before as separate genes (Olkkola *et al*., 2016) and later revised as *ant(6)‐Ie* (Hormeño *et al*., 2018). To avoid the issues of gene duplication and gene paralogues they are considered as separate genes in this study. The *bla*_OXA‐61_ gene was significantly more prevalent in *C. jejuni* (64.8%) than *C. coli* isolates (51.1%; Fisher's exact test; *p* < 0.05), while the *ant‐like* A gene was more prevalent in *C. coli* (40.22% of *C. coli* and 1.19% of *C. jejuni* isolates, *p* < 0.001). The prevalence of the *ant‐like* A gene was also significantly higher in multidrug resistant isolates (33.7%) compared to non‐multidrug resistant isolates (6.7%; *p* < 0.001; Fig. 2, Table 3), and associated (*p* < 0.005) with isolates from humans (14.5%) and sewage (13.3%) compared to those from animals (1.2%; *p* < 0.005; Fig. 2, Table 3). In the case of non‐multidrug resistant isolates, the frequency difference of the *ant‐like* A gene can probably be attributed to the frequency of *C. jejuni* in human infection samples compared to the abundance of *C. coli* from sewage. Genes associated with aminoglycoside resistance (*ant(6)‐Ia*, *sat‐4*, *ant(6)‐Ib*, *aad9*, *aph(3)‐IIIa*, *aph(2)‐IIIa*, *hpt* and *apmA*) were mainly found in *C. coli* multidrug resistant isolates while *sat‐1* was detected in only three *C. jejuni* strains from animals (Fig. 2, Table 3). Genes *ant(6)‐Ia*, *sat‐4*, *ant(6)‐Ib* and *aph(3)‐IIIa* were also found in *C. jejuni* isolates from animals (Fig. 2, Table 3). The *lnuC* gene, conferring resistance to lincosamides, was detected only in *C. coli* isolates and the *ermB* gene, which is not commonly found in *Campylobacter*, was detected in only one *C. coli* isolate from a chicken (Fig. 2, Table 3). A strong positive correlation (*p* < 0.001) between resistance phenotypes and genotypes was observed for tetracycline, streptomycin and gentamicin that were tested *in vitro* (Supporting Information Table S3). There was no correlation for erythromycin because the associated AMR gene *ermB* was only found in one isolate (Supporting Information Table S3). Concordance between putative resistance genotypes and laboratory phenotypes was lower than in some previous studies (Tyson *et al*., 2015; McDermott *et al*., 2016; Zhao *et al*., 2016). The main reason for this was that our study principally focused on the differential presence of AMR genes, to understand gene pool transmission, rather than resistance conferred by point mutation where it is more difficult to differentiate horizontal acquisition from *de novo* mutation. Other incongruences were observed between genotype prediction and laboratory phenotype. For example, not all isolates carrying aminoglycoside resistance genes were phenotypically resistant to streptomycin and gentamicin (Supporting Information Table S3). This is consistent with previous studies (Tyson *et al*., 2015; McDermott *et al*., 2016) and is potentially associated with variation in gene expression levels or synergistic effects among different resistance genes, warranting further study.

**Table 3:** Prevalence of 15 AMR genes in *Campylobacter jejuni* and *C. coli* isolates.^a^

	Multidrug resistant					Non‐multidrug resistant					Sensitive			Not tested		
	*Campylobacter jejuni* (*n* = 27)			*Campylobacter coli* (*n* = 56)		*Campylobacter jejuni* (*n* = 129)			*Campylobacter coli* (*n* = 35)		*Campylobacter jejuni* (*n* = 7)			*Campylobacter jejuni* (*n* = 5)^b^		*Campylobacter coli* (*n* = 1)^b^
	Animals (*n* = 17)	Humans (*n* = 10)	Animals (*n* = 11)	Humans (*n* = 18)	Sewage (*n* = 27)	Animals (*n* = 25)	Humans (*n* = 101)	Sewage (*n* = 3)	Humans (*n* = 15)	Sewage (*n* = 20)	Animals (*n* = 2)	Humans (*n* = 4)	Sewage (*n* = 1)	Humans (*n* = 3)	Sewage (*n* = 2)	Humans (*n* = 1)
*bla* OXA‐61	15/17 (88.24%)	6/10 (60.00%)	8/11 (72.73%)	9/18 (50.00%)	9/27 (33.33%)	16/25 (64.00%)	65/101 (64.36%)	1/3 (33.33%)	8/15 (53.33%)	13/20 (65.00%)	1/2 (50.00%)	3/4 (75.00%)	0/1 (0%)	1/3 (33.33%)	0/2 (0.00%)	0/1 (0.00%)
*tet* O	14/17 (82.35%)	4/10 (40.00%)	8/11 (72.73%)	16/18 (88.89%)	23/27 (85.19%)	20/25 (80.00%)	80/101 (79.21%)	1/3 (33.33%)	12/15 (80.00%)	16/20 (80.00%)	0/2 (0.00%)	0/4 (0/00%)	0/1 (0%)	3/3 (100.00%)	1/2 (50.00%)	1/1 (100.00%)
*ant‐like* B	0/17 (0.00%)	0/10 (0.00%)	1/11 (9.09%)	3/18 (16.67%)	2/27 (7.41%)	1/25 (4.00%)	1/101 (0.99%)	0/3 (0.00%)	0/15 (0.00%)	3/20 (15.00%)	0/2 (0.00%)	0/4 (0/00%)	0/1 (0%)	0/3 (0.00%)	0/2 (0.00%)	0/1 (0.00%)
*ant‐like* A	0/17 (0.00%)	0/10 (0.00%)	5/11 (45.45%)	12/18 (66.67%)	11/27 (40.74%)	1/25 (4.00%)	1/101 (0.99%)	0/3 (0.00%)	1/15 (6.67%)	8/20 (40.00%)	0/2 (0.00%)	0/4 (0/00%)	0/1 (0%)	0/3 (0.00%)	0/2 (0.00%)	0/1 (0.00%)
*ant(6) Ia*	4/17 (23.53%)	0/10 (0.00%)	1/11 (9.09%)	3/18 (16.67%)	6/27 (22.22%)	1/25 (4.00%)	0/101 (0.00%)	0/3 (0.00%)	1/15 (6.67%)	0/20 (0.00%)	0/2 (0.00%)	0/4 (0/00%)	0/1 (0%)	0/3 (0.00%)	0/2 (0.00%)	1/1 (100.00%)
*sat‐4*	4/17 (23.53%)	0/10 (0.00%)	2/11 (18.18%)	3/18 (16.67%)	5/27 (18.52%)	1/25 (4.00%)	0/101 (0.00%)	0/3 (0.00%)	1/15 (6.67%)	0/20 (0.00%)	0/2 (0.00%)	0/4 (0/00%)	0/1 (0%)	0/3 (0.00%)	0/2 (0.00%)	1/1 (100.00%)
*lnuC*	*0/17* (0.00%)	0/10 (0.00%)	1/11 (9.09%)	0/18 (0.00%)	3/27 (11.11%)	0/25 (0.00%)	0/101 (0.00%)	0/3 (0.00%)	1/15 (6.67%)	4/20 (20.00%)	0/2 (0.00%)	0/4 (0/00%)	0/1 (0%)	0/3 (0.00%)	0/2 (0.00%)	0/1 (0.00%)
*ant(6)*‐Ib	5/17 (29.41%)	1/10 (10.00%)	5/11 (45.45%)	4/18 (22.22%)	8/27 (29.63%)	0/25 (0.00%)	0/101 (0.00%)	0/3 (0.00%)	0/15 (0.00%)	0/20 (0.00%)	0/2 (0.00%)	0/4 (0/00%)	0/1 (0%)	0/3 (0.00%)	0/2 (0.00%)	0/1 (0.00%)
*aad9*	0/17 (0.00%)	1/10 (10.00%)	4/11 (36.36%)	5/18 (27.78%)	8/27 (29.63%)	0/25 (0.00%)	0/101 (0.00%)	0/3 (0.00%)	0/15 (0.00%)	0/20 (0.00%)	0/2 (0.00%)	0/4 (0/00%)	0/1 (0%)	0/3 (0.00%)	0/2 (0.00%)	0/1 (0.00%)
*aph(3)‐IIIa*	2/17 (11.76%)	0/10 (0.00%)	0/11 (0.00%)	2/18 (11.11%)	1/27 (3.7%)	1/25 (4.00%)	0/101 (0.00%)	0/3 (0.00%)	1/15 (6.67%)	0/20 (0.00%)	0/2 (0.00%)	0/4 (0/00%)	0/1 (0%)	0/3 (0.00%)	0/2 (0.00%)	1/1 (100.00%)
*aph(2)‐IIIa*	0/17 (0.00%)	0/10 (0.00%)	2/11 (18.18%)	1/18 (5.56%)	4/27 (14.81%)	0/25 (0.00%)	0/101 (0.00%)	0/3 (0.00%)	0/15 (0.00%)	0/20 (0.00%)	0/2 (0.00%)	0/4 (0/00%)	0/1 (0%)	0/3 (0.00%)	0/2 (0.00%)	0/1 (0.00%)
*hyg*	0/17 (0.00%)	0/10 (0.00%)	1/11 (9.09%)	2/18 (11.11%)	6/27 (22.22%)	0/25 (0.00%)	0/101 (0.00%)	0/3 (0.00%)	0/15 (0.00%)	0/20 (0.00%)	0/2 (0.00%)	0/4 (0/00%)	0/1 (0%)	0/3 (0.00%)	0/2 (0.00%)	0/1 (0.00%)
*apmA*	0/17 (0.00%)	1/10 (10.00%)	1/11 (9.09%)	0/18 (0.00%)	0/27 (0.00%)	0/25 (0.00%)	0/101 (0.00%)	0/3 (0.00%)	0/15 (0.00%)	0/20 (0.00%)	0/2 (0.00%)	0/4 (0/00%)	0/1 (0%)	0/3 (0.00%)	0/2 (0.00%)	0/1 (0.00%)
*sat‐1*	3/17 (17.65%)	0/10 (0.00%)	0/11 (0.00%)	0/18 (0.00%)	0/27 (0.00%)	0/25 (0.00%)	0/101 (0.00%)	0/3 (0.00%)	0/15 (0.00%)	0/20 (0.00%)	0/2 (0.00%)	0/4 (0/00%)	0/1 (0%)	0/3 (0.00%)	0/2 (0.00%)	0/1 (0.00%)
*ermB*	0/17 (0.00%)	0/10 (0.00%)	1/11 (9.09%)	0/18 (0.00%)	0/27 (0.00%)	0/25 (0.00%)	0/101 (0.00%)	0/3 (0.00%)	0/15 (0.00%)	0/20 (0.00%)	0/2 (0.00%)	0/4 (0/00%)	0/1 (0%)	0/3 (0.00%)	0/2 (0.00%)	0/1 (0.00%)

a Isolates are separated as multidrug or non‐multidrug resistant based on their *in vitro* phenotypic profile.

b Isolates id: 5087, 5093, 5111, 5095, 5100, 5215 were not tested for antibiotic resistant profile *in vitro*.

### *AMR genes are co‐localized in the genome of multidrug resistant isolates*

**Figure 3:**
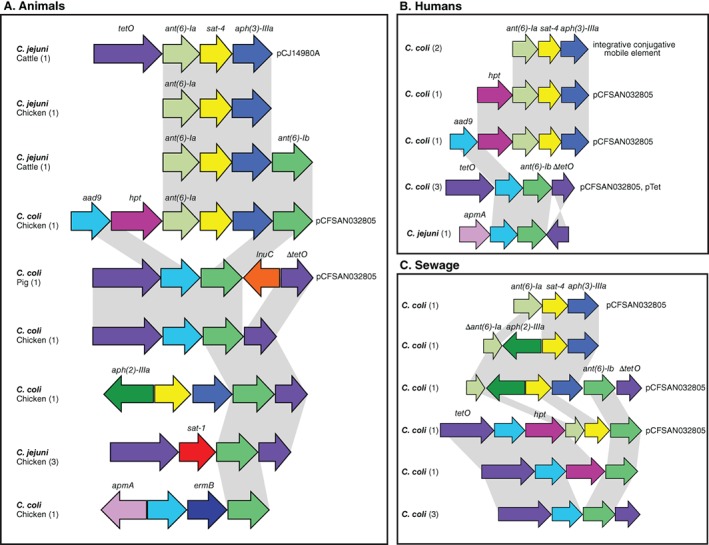
Comparative genetic organization of AMR GIs in *Campyloabcter*. The presence of each AMR gene, highlighted in different colours, is shown for representative *C. jejuni* and *C. coli* isolate genomes sampled from animals (A), humans (B) and sewage (C). The number of isolate genomes containing each genomic island arrangement is indicated in the parenthesis. Grey shading identifies sequence that shares > 95% nucleotide sequence identity. The name of the plasmid or mobile genetic element, associated with each genomic island, is indicated. [Color figure can be viewed at http://wileyonlinelibrary.com]

AMR genes are often found in close proximity in the genome. For example, aminoglycoside resistance genes can form localized clusters within the genome (Werner *et al*., 2003; Qin *et al*., 2012). The low numbers of *apmA* and *ermB* genes identified, excluded them from formal statistical comparison. Due to the high levels of resistance to fluoroquinolones and tetracycline, the presence of *ant‐like* A, *ant(6)‐Ia*, *sat‐4*, *ant(6)‐Ib*, *aad9*, *aph(3)‐IIIa*, *aph(2)‐IIIa*, *sat‐1* and *hpt* genes, was by definition significantly associated with MDR (Fisher's exact test; *p* < 0.001), because this was defined as resistance to three or more antimicrobial classes (Table 3). There was a slight increasing trend in the presence of *ant‐like* A, *ant‐like* B, *aad9*, *ant(6)‐Ia*, *sat‐4*, *ant(6)‐Ib* and *aph(3)‐IIIa* genes from 2010 to 2015 (Supporting Information Fig. S1). Furthermore, the relative position of the 15 AMR genes (in contiguous sequence assemblies) detected in *Campylobacter* isolates revealed two types of genetic associations in animal, human and sewage isolates. The first was between *ant(6)‐Ia*, *sat‐4* and *aph(3)‐IIIa* genes, which clustered together in three *C. jejuni* isolates (one from chicken and two from cattle) and in eight *C. coli* isolates (one from chicken, four from humans and three from sewage; Fig. 3). This cluster has been previously described with the three genes located on the same genomic island in *C. coli* (Qin *et al*., 2012). The further addition of the *aph(2)‐IIIa* gene to this genomic island was observed in two *C. coli* isolates from sewage (Fig. 3). The second type of genetic association involved the presence of *tetO*, *aad9* and *ant(6)‐Ib* genes. These genes clustered together in six *C. coli* isolates (one from chicken, one from pig, one from human and three from sewage) but also in one *C. jejuni* isolate from a human patient (Fig. 3). The addition of the *sat‐1*, *hpt*, *apmA* and *ermB* genes was also observed in these two types of syntenic block (Fig. 3).

### *Evidence of gene pool transmission AMR genes*

Evidence for HGT has been demonstrated for AMR genes in various bacteria, including *Campylobacter* (Sheppard *et al*., 2011, 2013; Wang *et al*., 2014; Sheppard and Maiden, 2015; Li *et al*., 2017), in some cases facilitated by mobile genetic elements including plasmids and transposons (Boerlin and Reid‐Smith, 2008). We identified one plasmid (pCFSAN032805; Accession: CP023546.1) in the genome sequences of eight *C. coli* isolates (one from chicken, one from a pig, three from humans and three from sewage; Fig. 3). Furthermore, a *C. jejuni* plasmid (pCJ14980A; Accession: CP017030.1) previously isolated from turkey faeces (Florez‐Cuadrado *et al*., 2017) was identified in a *C. jejuni* isolate from cattle in our study (Fig. 3). A pTet plasmid (Accession: CP002030.1) was also detected in one *C. coli* isolate of human origin (Fig. 3). A genomic region that was carrying the gene cluster *ant(6)‐Ia*, *sat‐4* and *aph(3)‐IIIa* was highly similar to an integrative conjugative mobile element described in *Erysipelothrix rhusiopathiae* (Accession: MG812141.1) isolated from a pig farm. This region was also similar to sequences from other bacteria like *Clostridium difficile*, *Staphylococcus aureus*, *S. pseudintermedius*, *Streptococcus suis* and *Enterococcus faecium*. These findings are consistent with the circulation of genes, and more specifically alleles, not only between host microbiome gene pools but also between *Campylobacter* species. To investigate this further, we compared allelic diversity for the 15 identified AMR genes in *C. jejuni* and *C. coli* isolates.

The genes, *bla*_OXA‐61_ and *tetO*, had the highest diversity with 34 and 47 different alleles detected in *C. jejuni* and in *C. coli* isolates respectively (Fig. 3, Supporting Information Fig. S1). There were five *bla*_OXA‐61_ alleles, two of which were present in 16 and four *C. jejuni* and in 50 and five *C. coli* isolates, respectively (Fig. 3, Supporting Information Fig. S1). For the *tetO* gene, six alleles were present in more than five isolates each, with the most common allele present in 19 *C. jejuni* and in 35 *C. coli*. For the *aad9* and *ant(6)‐Ib* gene, both of which had five alleles, the most common allele was present in both *C. jejuni* and *C. coli* isolates from multiple sources (Fig. 3, Supporting Information Fig. S1, Table S2). Finally, the *sat‐4* gene shared two out of the six alleles between four *C. jejuni* and four *C. coli* isolates and the *apmA* gene had one allele which was shared by a *C. jejuni* of human origin and a *C. coli* isolated from a chicken (Fig. 3, Supporting Information Fig. S1, Table S2). Remaining alleles were detected exclusively in *C. coli* isolates.

### *Clonal descent is disrupted in AMR genes*

**Figure 4:**
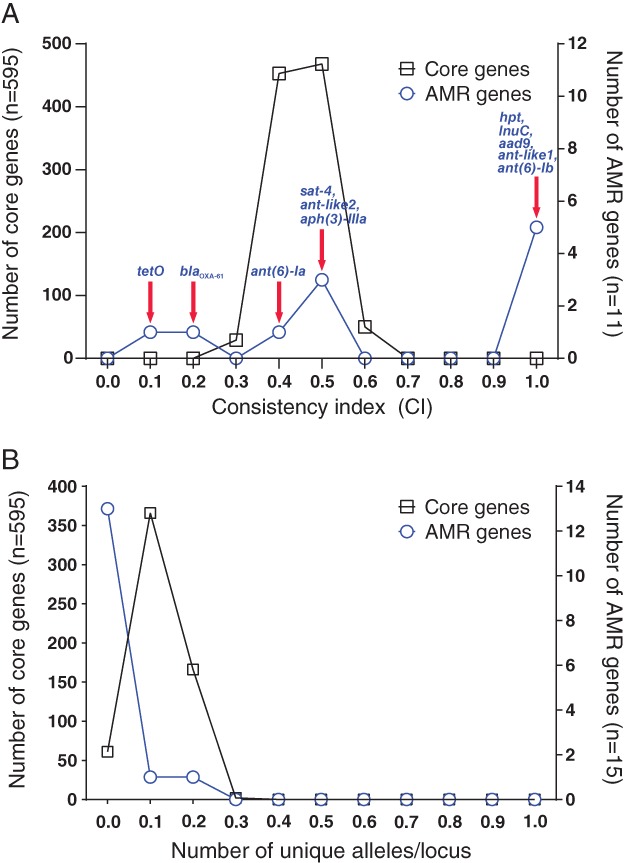
Comparison of consistency index and allelic variation between AMR and core genes. A. Consistency indices to a core phylogeny, were calculated for each gene alignment for AMR and core genes using the *phangorn* package in R. B. The number of alleles per locus. The left *y*‐axis indicates the number of core genes (black line), the right *y*‐axis indicates the number of AMR genes (blue line). For the consistency index, the two distributions were significantly different (two‐tailed Mann–Whitney test; *p* = 0.0214, Mann–Whitney *U* = 3307). For the number of alleles per locus, the two distributions were significantly different (two‐tailed Mann–Whitney test; *p* < 0.0001, Mann–Whitney *U* = 1004). [Color figure can be viewed at http://wileyonlinelibrary.com]

The mean consistency index (CI) was significantly higher (Mann–Whitney test; *U* = 3307, *p* = 0.0214) among AMR genes (0.65581 ± 0.3531) compared with 595 core genes (0.4552 ± 0.05799; Fig. 4A). This provides evidence that the clonal mode of descent has been disrupted in AMR genes consistent with HGT. Furthermore, there was a significant decrease in the average allelic variation among AMR genes compared to core genes (Mann–Whitney test; *U* = 1004, *p* ≤ 0.0001; Fig. 4B). The average number of unique alleles per isolate was 0.03436 ± 0.05218 for the 15 AMR genes, compared with 0.1169 ± 0.05248 for 595 core genes. This is consistent with HGT facilitating the movement of AMR genes into multiple genetic backgrounds.

**Figure 5:**
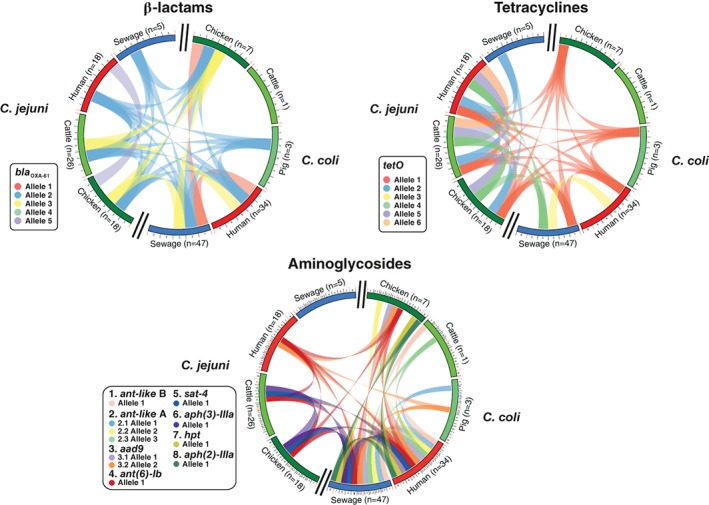
Distribution of AMR gene alleles among *Campylobacter* species and isolate source. Circus plots indicate the number of *C. jejuni* and *C. coli* isolates sampled from chickens (dark green), cattle (green), pigs (light green), humans (red) and sewage (blue) that contain genes associated with resistance to β‐lactam, tetracycline and aminoglycoside antimicrobials. Alleles present in > 5 isolate genomes are numbered around the perimeter. Exact matches between allele sequences are indicated by joining lines, coloured differently for different alleles. [Color figure can be viewed at http://wileyonlinelibrary.com]

Among the AMR genes present in five or more isolates, the *bla*_OXA‐61_ and *tetO* alleles, associated with resistance to β‐lactams and tetracyclines respectively, were almost ubiquitous among *C. jejuni* and in *C. coli* from different sources. Two common *bla*_OXA‐61_ alleles were present in both *Campylobacter* species in all different hosts and sewage with other alleles shared only between human, chicken and sewage isolates (Fig. 5). A single *tetO* allele was present in the genomes of isolates from all different hosts and sewage except for *C. jejuni* from humans and *C. coli* cattle (Fig. 5), possibly due to low sample numbers (Supporting Information Table S1). Another *tetO* allele was shared between *C. coli* isolates from sewage and *C. jejuni* from chickens, cattle and humans (Fig. 5). In addition to evidence of frequent allele sharing between *Campylobacter* species from multiple sources, there were also several species‐specific alleles found in isolates from multiple sources (Fig. 5). AMR genes associated with aminoglycoside resistance had less allelic diversity compared to *bla*_OXA‐61_ and *tetO* (Fig. 2) and showed evidence of gene pool transmission between bacterial species and isolate source populations. Three alleles of the *aad9*, *ant(6)‐Ib*, *sat‐4* genes were shared between *C. jejuni* and *C. coli* isolates. The *ant(6)‐Ib* allele was found in *C. jejuni* isolates from humans, cattle, chickens and in *C. coli* isolates from humans, chickens and sewage. The *aad9* allele was found in human *C. jejuni* isolates and in *C. coli* isolates from humans, chickens, pigs and sewage. The *sat‐4* allele was found in *C. jejuni* isolates from cattle and chicken and in *C. coli* isolates from human, chicken and sewage sources (Fig. 5). Alleles of other genes associated with aminoglycoside resistance (*ant‐like* A, *aad9*, *ant(6)‐Ib*, *aph(3)‐IIIa*, *hpt* and *aph(2)‐IIIa*) also showed evidence of transfer (allele sharing) between isolates sampled from different sources (Fig. 5).

## Discussion

Forecasts of rising AMR in bacteria can make dramatic claims, such as an associated death toll of 10 million people by 2050 if no action is taken (Balouiri *et al*., 2016). However, for priority pathogens such as *Campylobacter* (WHO, 2017) it is not always clear where such action should be targeted. One reason for this is that zoonotic bacteria do not reside in a single host niche, therefore the source and sink dynamics of resistant strains may be poorly understood. Furthermore, the conduit for transmission between niches (in this case faeces) may also represent a reservoir of AMR. Here, by focussing analyses on comparison of gene pools, rather than individual resistant clones, we directly address if the alleles that confer resistance have spread between pathogenic *Campylobacter* species and the niches in which they reside.

Human infection is often a dead‐end for *Campylobacter* as disease is usually self‐limiting and human‐to‐human transmission is uncommon. As antibiotic treatment for campylobacteriosis is generally only given in acute or persistent cases, the heavy use of related antimicrobials in human and veterinary medicine (Schwarz *et al*., 2001; Teuber, 2001; Livermore, 2007) has raised concerns about how selection for resistance in livestock may lead to AMR in human pathogens. Despite the ban on the use of antibiotics as growth promoters in animals in 2006, quinolones and tetracyclines are still available for treatment of livestock all over the world (WHO, 2017). Consistent with trends in a recent ECDC report (Food and Authority, 2019), resistance to ciprofloxacin and tetracycline was seen in both *Campylobacter* species in our study, with resistance to streptomycin and gentamicin also frequent among sequenced *C. coli* isolates (Table 1). This may not be surprising as Spain has the highest sale of aminoglycosides for veterinary use in the EU (European Medicines Agency, 2018). Perhaps equally worrying was the isolation of *C. coli* resistant to erythromycin which is the drug of choice for antibiotic treatment of clinical campylobacteriosis (Acheson and Allos, 2001). The extent to which this level of resistance is a legacy of past use of fluoroquinolones, tetracyclines (Toth *et al*., 2013; Cameron and Gaynor, 2014) and other antimicrobials is not known but it is clear that *Campylobacter* harbour numerous resistance genes, potentially exacerbated by the carriage of similar genes among other components of the microbiota (van den Bogaard, 2000; Holmes *et al*., 2016).

AMR is widespread among *Campylobacter* isolated from livestock (Qin *et al*., 2014; Wang *et al*., 2014; Florez‐Cuadrado *et al*., 2016; Sproston *et al*., 2018), but the transmission dynamics are poorly understood. Where resistance is conferred by a single (or few) nucleotide substitution(s), such as in the *gyrA* gene (fluoroquinolone resistance; Engberg *et al*., 2001; Payot *et al*., 2006; Zhao *et al*., 2016), it is impossible to tell from sequence data if HGT or point mutation were responsible. For other classes of antibiotics, for example tetracyclines, there is evidence for the transfer of genes (e.g., *tetO*) between *C. jejuni* isolates, even in the absence antimicrobial selective pressure (Qin *et al*., 2012). In addition to *tetO*, our analyses identified 14 other accessory genes associated with *Campylobacter* resistance to other known antimicrobial classes (Supporting Information Table S4). These included aminoglycosides (10 genes), β‐lactams (*bla*_OXA‐61_) and macrolides (*erm*B) that have been variously used as treatments targeting *Campylobacter* and other infectious agents (or even as growth promoters; Engberg *et al*., 2001) in humans and animals (Lambert *et al*., 1985; Engberg *et al*., 2001; Griggs *et al*., 2009; Qin *et al*., 2012, 2014; Chen *et al*., 2013; Toth *et al*., 2013; Florez‐Cuadrado *et al*., 2016, 2017; Lapierre *et al*., 2016; Yao *et al*., 2017). Initial evidence of the importance of HGT in the transmission of these genes can be seen with inconsistent topology of individual AMR gene trees, compared to the *Campylobacter* core genome phylogeny (Supporting Information Fig. S1). Specifically, the CI varied for the 11 AMR genes, highlighting a disparity in the amount of inferred homoplasy in these genes, compared to genes in the core genome (Fig. 4B). Furthermore, the allelic variation in the AMR‐associated genes was significantly lower than the mean for core genes. Convergent genotypes may have evolved multiple times in different genetic backgrounds, however the most parsimonious explanation is the spread of AMR via HGT.

Perhaps the most compelling evidence for HGT is the identification of co‐localized clusters of genes that constitute GIs. Consistent with evidence of aminoglycoside resistance in *Campylobacter* (Lambert *et al*., 1985; Gibreel *et al*., 2004; Qin *et al*., 2012; Lapierre *et al*., 2016), all AMR genes detected in our study were found in MDR GIs, except for bla_OXA‐61_, *ant‐like* A and *ant‐like* B. There were multiple syntenic arrangements of genes with some GIs containing genes that confer resistance to more than one antimicrobial drug class (macrolides and aminoglycosides) as previously reported (Werner *et al*., 2003). Some of the MDR GIs are known from previous studies (*ant(6)‐Ia*, *sat‐4* and *aph(3)‐IIIa*; Derbise *et al*., 1996, 1997), while others are reported here for the first time, such as the association between *TetO*, *aad9* and *ant(6)‐Ib* genes. GI similarities provide evidence of transfer between *C. jejuni* and *C. coli*, and gene pool transmission among isolates from animals, humans and sewage. The transfer of GIs in *Campylobacter* can be via natural transformation (Qin *et al*., 2012), however several GIs were found on plasmids or integrative conjugative elements (Fig. 3) indicating the active mobilization of gene clusters. GIs containing the *ant(6)‐Ia*, *sat‐4* and *aph(3)‐IIIa* cluster, and the *tetO* gene, have previously been described in staphylococci (Lambert *et al*., 1985; Derbise *et al*., 1996, 1997). Furthermore, the conjugative transposon found in *C. coli* was highly similar (~99.4% nucleotide identity over at least 60% of the sequence) to related sequence in other Gram‐positive bacteria. This is consistent with the circulation of AMR genes not only among *Campylobacter* species in different habitats but also HGT from other bacteria (Trieu‐Cuot *et al*., 1985; Zilhao *et al*., 1988).

An important finding in our study was that *C. coli* carry more combinations of AMR genes simultaneously than *C. jejuni* (Table 2). A simple explanation could be that *C. coli* ST‐828 complex isolates are more recombinogenic. There is evidence of the accumulation of *C. jejuni* DNA throughout the genome of this lineage (Sheppard *et al*., 2008, 2013) which could have led to the acquisition of multiple AMR genes. It is also possible that the dominance of this *C. coli* lineage (ST‐828 complex), that is much less diverse than *C. jejuni* as a whole, reflects a genetic bottleneck that favoured an ancestral AMR strain in, for example, the pig gut where *C. coli* (Thakur *et al*., 2006) and antimicrobial exposure (Aarestrup *et al*., 2000) are common. Whatever the reasons for differences in MDR between *C. jejuni* and *C. coli*, there is clear evidence for HGT and the transmission of AMR genes among bacterial species and host niche gene pools.

Contrasting evidence of HGT with quantitative information about the transmission of resistant bacteria between hosts would be extremely useful for understanding the dissemination of AMR among isolates from different habitats. In *Campylobacter*, studies have attempted to estimate the number of strains excreted into the environment by different animals (Ogden *et al*., 2009) and attribute the source of human infection to livestock (especially poultry) reservoirs (Sheppard *et al*., 2009b; Thépault *et al*., 2017, 2018). However, these large‐scale probabilistic studies are utterly underpowered for investigating the almost infinite number of possible transmission events, where the survival and proliferation of a single strain in a new niche could lead to the transfer of AMR genes between hosts and environments. A theoretical solution to the spread of AMR could be to use different drug classes in animals on the assumption that distinct antimicrobial selection pressures would sustain efficacy of drugs in humans. However, even if this was feasible, evidence from this study (and others (Hendriksen *et al*., 2019)) shows that multidrug resistant bacteria can be isolated and cultured from sewage, presenting a potential route for transmission of AMR in the environment. While the sources and implications of environmental contamination remain controversial (Rizzo *et al*., 2013; Munck *et al*., 2015), the evidence in our study is consistent with the horizontal transfer of AMR among *Campylobacter* isolated from livestock, humans and sewage. This suggests that judicious use of antimicrobials and monitoring of the amount of AMR *Campylobacter* entering the environment may be beneficial in combating the rise of resistance in this important zoonotic pathogen.

## Experimental procedures

### *Culture and antimicrobial susceptibility testing*

As part of routine *Campylobacter* surveillance in Spain, isolates were sampled and cultured on blood agar plates (bioMérieux) and incubated for 48 h at 37°C under microaerophilic conditions using Campygen atmosphere generation system packs (Oxoid, Basingstoke, U.K.). Subcultured colonies were harvested and suspended in sterile water to a standardized cell density (0.5 McFarland turbidity). Fifty microliters of this suspension was added to 11 ml of Mueller‐Hinton broth (TREK Diagnostics Systems, Waltham, MA) supplemented with 5.5% lysed horse blood (Oxoid). The solution was poured onto EUCAMP2 microdilution plates (TREK Diagnostics Systems) which were incubated under microaerophilic conditions for 48 h at 37°C as previously described (Florez‐Cuadrado *et al*., 2017). The interpretation of the quantitative data was performed according to the European Committee of Antimicrobial Susceptibility Testing, EUCAST (http://www.eucast.org/; last accessed: 06/2017).

### *DNA extraction, genome sequencing and archiving*

A total of 260 *Campylobacter* isolates (167 *C. jejuni* and 92 *C. coli*) that displayed MDR phenotypes were chosen for genome sequencing. These represented strains sampled from humans, livestock and urban effluents in Spain. Of these, 55 isolates originated in animals (44 *C. jejuni* and 11 *C. coli*) including broiler chickens (18 *C. jejuni* and 7 *C. coli*), cattle (26 *C. jejuni* and 1 *C. coli*) and pigs (3 *C. coli*) and were collected from abattoirs in Spain (2008–2011) as part of the Spanish Veterinary Antimicrobial Resistance Surveillance (VAV) Network (Supporting Information Table S1). The isolates were chosen on the basis of resistance profiles (susceptible to resistant) to five different antibiotics (Table 1). Human samples (*n* = 152; 118 *C. jejuni* and 34 *C. coli*) were associated with campylobacteriosis cases in hospitals in the regions of Castilla y Leon, Extremadura and Andalucía between 2013 and 2016. *Campylobacter* isolates of urban effluent origin (*n* = 53; 6 *C. jejuni* and 47 *C. coli*) were collected from the wastewater treatment plants in the city of Madrid (Spain) between 2011 and 2013 (Ugarte‐Ruiz *et al*., 2015). All isolates were obtained using culture based methods (Moreno *et al*., 2000; Ugarte‐Ruiz *et al*., 2015; Hormeño *et al*., 2016) and speciated as *C. jejuni* or *C. coli* using a conventional multiplex PCR as previously described (Ugarte‐Ruiz *et al*., 2012).

For genome sequencing, isolates stored at −80°C in 1% protease peptone and 10% glycerol broth were cultured onto blood agar plates (bioMérieux) in microaerophilic conditions at 42°C for 48 h as previously described (Florez‐Cuadrado *et al*., 2017). Genomic DNA was extracted using the QIAamp DNA Mini Kit (QIAGEN, Crawley, U.K.), according to manufacturer's instructions. Nucleic acid content was quantified on a Nanodrop spectrophotometer prior to normalization and sequencing. Libraries were prepared with Nextera XT kits (v2) and high‐throughput sequencing was performed using an Illumina MiSeq sequencer (Illumina, San Diego, CA; v3 technology, 300 bp paired‐end). Short reads were assembled *de novo* using SPAdes (version 3.8.0). All genomes used in this study were archived on the BIGSdb web‐based database platform (Jolley and Maiden, 2010) and given a unique identification number (BIGSid; Supporting Information Table S1).

### *Phylogenetic analysis*

A pangenome was created for all isolate genomes in our collection as the sum of core genes, shared by all isolates, and accessory genes, present in at least one isolate. Genomes with a total assembly length > 1.9 Mbp, > 500 contigs or an N_95_ < 800 bp were considered poor quality and were excluded from the phylogenetic analyses. Whole genome multiple sequence alignments were obtained using MAFFT (Katoh, 2002) following a gene‐by‐gene approach as previously described (Méric *et al*., 2014). Phylogenetic trees, based on gene‐by‐gene alignments of core genes (Méric *et al*., 2014) or single gene sequences, were reconstructed using the Neighbour joining clustering method (Saitou and Nei, 1987).

### *Screening for AMR genes*

AMR genes were identified in all *Campylobacter* genomes by comparison with the CARD (Jia *et al*., 2017; last assessed: 03/06/2017), the ResFinder (Zankari *et al*., 2012) and the NCBI databases using the BLAST algorithm (Sheppard *et al*., 2012; Maiden *et al*., 2013). A locus match was defined when genes had > 70% nucleotide identity over > 50% of the sequence length, and a matrix was generated that contained presence/absence information for each card gene and the allelic variation at that locus for every genome. Following the identification of isolate genomes harbouring one or more AMR genes, contigs were screened for upstream and downstream open reading frames (ORFs) to characterize the location of AMR relative to adjacent genes, using SnapGene software (GSL Biotech; available at http://snapgene.com). A second confirmatory analysis was performed, in which contigs were compared to NCBI database to identify whether they are associated with known plasmid or mobile elements. Sequence matches with > 95% nucleotide identity over > 50% of the sequence length were considered positive hits. A bivariate analysis was performed, in Stata version 14.0 (StataCorp, College Station, TX), to determine the relationship between phenotypes and genotypes for the presence of resistance using the Fisher's exact test. Associations were considered significant when *p* < 0.05.

### *HGT among infection‐associated genes*

Population genetic analyses were undertaken to compare molecular variation among AMR genes to investigate patterns of HGT between species and isolates sampled form different niches. Genes where AMR is mediated by single nucleotide polymorphisms (SNPs), for example *gyr*A in fluroquinolone resistance (Sproston *et al*., 2018), were excluded from this analysis because of the inability to distinguish *de novo* mutation from homologous recombination of similar sequence. The allelic variation was calculated at loci associated with AMR genes (*n* = 15) and compared to variation at core loci (*n* = 595 genes). For both groups, the number of alleles at each locus (determined using a whole‐genome multilocus sequence typing, MLST, approach (Sheppard *et al*., 2012) and CI) were calculated. The consistency of a phylogenetic tree to patterns of variation in sequence alignments was determined for each gene of interest, and constituted an inference of the minimum amount of homoplasy in these genes, as implied by the tree (Kluge and Farris, 1969). The CI function from the R Phangorn package (Schliep, 2011) was used to calculate consistency indices for every single‐gene alignment of the 15 AMR genes to a phylogeny constructed from a concatenated gene‐by‐gene alignment of 595 core genes shared by all 259 isolates. The average CI of these shared genes was compared to that of the AMR genes.

## Supporting information


**Table S1**. Details of isolates used in this study.

**Table S2**. Isolates and their MIC against different antibiotics used in this study.

**Table S3**. Resistance phenotype–genotype correlations among *Campylobacter* isolates.

**Table S4**. Antibiotic drug classes: mechanism of action/resistance and AMR genes.

**Table S5**. Genomic and phenotypic details of all isolates used in this study.

**Fig. S1**. Individual AMR gene trees. 14 single‐gene trees highlighting the allelic diversity in AMR genes found in *C.jejuni* (grey) and *C.coli* (black) isolates shown in the first column. The resistance status of each isolate is highlighted in the second column for multidrug resistant (dark pink), non‐multidrug resistance (light pink) or not tested (white). The host of every isolate is shown in the third column for chickens (dark green), cattle (green), pigs (light green), humans (red) and sewage (blue). The scale bars represent the number of substitutions per site.

**Fig. S2**. Prevalence of AMR genes over time. Graphs illustrate the presence of 15 putative AMR genes in isolate genomes sampled at each year in the study. Prevalence (%) was calculated by dividing the number of samples that had the AMR gene by the total number of samples in that year.

## Data Availability

All sequence data are linked to NCBI BioProject PRJNA528879. The bacterial genomes are available in GenBank under accession codes SRX5575129 to SRX5587545.
