# Supplementary material for: Gene pool transmission of multidrug resistance among Campylobacter from livestock, sewage and human disease
Source: Environ Microbiol. 2019 Aug 27;21(12):4597–613. doi: 10.1111/1462-2920.14760 (PMC6916351; doi:10.1111/1462-2920.14760)
Supplement: Supplementary file 1 — Table S1. Details of isolates used in this study. [file EMI-21-4597-s003.pdf]

Table S1. Details of isolates used in this study

Id	Strain name	ZTA ID	Year of isolation	Species	Source	Sequence type (ST)	ST - complex	Genome size	Number of contigs	Used in phylogentic analysis	Resistance status	Biosample accession number
5040	D.01	ZTA10/02396 CPFA Preston	2010	*C. coli*	Sewage	825	ST-828	1565712	555	Y	Multidrug resistant	SRR8785326
5041	D.03	ZTA11/00429 CPD Preston	2011	*C. coli*	Sewage	825	ST-828	1657080	49	Y	Multidrug resistant	SRR8785323
5042	D.06	ZTA11/01227 CPF Preston	2011	*C. coli*	Sewage	825	ST-828	1648170	124	Y	Multidrug resistant	SRR8785324
5043	D.07	ZTA11/01965 CPD Preston	2011	*C. coli*	Sewage	825	ST-828	1702908	61	Y	Multidrug resistant	SRR8785331
5044	D.08	ZTA11/01965 CPF Preston	2011	*C. coli*	Sewage	1055	ST-828	1603157	585	Y	Multidrug resistant	SRR8785332
5045	D.09	ZTA11/02394 CPD Preston	2011	*C. coli*	Sewage	962	ST-828	1690424	70	Y	Non-multidrug resistant	SRR8785307
5046	D.10	ZTA11/02394 CPF Preston	2011	*C. coli*	Sewage	1107	ST-828	1684730	84	Y	Non-multidrug resistant	SRR8785306
5047	D.11	ZTA11/03024-2 CPF Preston	2011	*C. coli*	Sewage	829	ST-828	1651636	153	Y	Non-multidrug resistant	SRR8785309
5048	D.12	ZTA11/03517-3 CPD Preston estría	2011	*C. coli*	Sewage	3246	ST-828	1627190	181	Y	Multidrug resistant	SRR8785308
5050	D.14	ZTA11/03517-4 CPF Preston	2012	*C. coli*	Sewage	825	ST-828	1647683	83	Y	Non-multidrug resistant	SRR8785302
5051	D.15	ZTA12/00985 CPF Preston Filtro	2012	*C. coli*	Sewage	1055	ST-828	1629124	469	Y	Multidrug resistant	SRR8785305
5052	D.16	ZTA12/01820 CPDA Preston	2012	*C. coli*	Sewage	899	ST-828	1639899	225	Y	Multidrug resistant	SRR8785304
5053	D.17	ZTA12/01820 CPFA Preston	2012	*C. coli*	Sewage	899	ST-828	1668423	158	Y	Multidrug resistant	SRR8785311
5054	D.18	ZTA12/01820 CPFB Preston	2012	*C. coli*	Sewage	899	ST-828	1649812	151	Y	Multidrug resistant	SRR8785310
5056	D.20	ZTA12/02041 CASAA Preston	2012	*C. coli*	Sewage	2097	ST-828	1760519	86	Y	Non-multidrug resistant	SRR8785471
5057	D.21	ZTA12/02041CASA A Filtro Preston	2012	*C. coli*	Sewage	1055	ST-828	1690694	98	Y	Multidrug resistant	SRR8785472
5058	D.22	ZTA12/02041 CASA B Preston	2012	*C. coli*	Sewage	2097	ST-828	1776891	108	Y	Non-multidrug resistant	SRR8785473
5059	D.23	ZTA12/02041 CASA B Preston Filtro	2012	*C. coli*	Sewage	1055	ST-828	1689977	59	Y	Multidrug resistant	SRR8798686
5060	D.24	ZTA12/02041 CASA C Preston	#N/A	*C. coli*	Sewage	825	ST-828	1645718	114	Y	Multidrug resistant	SRR8798685
5061	D.25	ZTA12/02041 CASA C Preston Filtro	2012	*C. coli*	Sewage	1055	ST-828	1580937	596	Y	Multidrug resistant	SRR8798688
5062	D.26	ZTA12/02041 CPFA Preston Filtro	2012	*C. coli*	Sewage	2097	ST-828	1757462	129	Y	Non-multidrug resistant	SRR8798687
5063	D.27	ZTA12/02041CPFB PRESTON FILTRO	2012	*C. coli*	Sewage	2097	ST-828	1352995	1106	Y	Non-multidrug resistant	SRR8798682
5064	D.28	ZTA12/02464 CASA Bolton	2012	*C. coli*	Sewage	825	ST-828	1663921	211	Y	Non-multidrug resistant	SRR8798681
5065	D.29	ZTA12/02464 CASA A Preston	2012	*C. coli*	Sewage	2077	ST-828	1660006	117	Y	Multidrug resistant	SRR8798684
5066	D.31	ZTA12/02464 CPD Preston	2012	*C. coli*	Sewage	825	ST-828	1675041	81	Y	Multidrug resistant	SRR8798647
5067	D.32	ZTA12/02464 CPFA Preston	2012	*C. coli*	Sewage	825	ST-828	1688967	68	Y	Non-multidrug resistant	SRR8798680
5068	D.33	ZTA12/02579 CASA Filtro	2012	*C. coli*	Sewage	860	ST-828	1794351	110	Y	Non-multidrug resistant	SRR8798698
5069	D.34	ZTA12/02579 CPD Preston	2012	*C. coli*	Sewage	825	ST-828	1693985	105	Y	Multidrug resistant	SRR8798642
5070	D.35	ZTA12/02579 CPD PRESTON	2012	*C. coli*	Sewage	826	ST-828	1686853	112	Y	Multidrug resistant	SRR8798641
5072	D.37	ZTA12/02785 CASA Filtro	2012	*C. coli*	Sewage	827	ST-828	1663188	99	Y	Multidrug resistant	SRR8798640
5073	D.38	ZTA12/02785 CASA Preston	2012	*C. coli*	Sewage	827	ST-828	1642583	228	Y	Multidrug resistant	SRR8798639
5074	D.39	ZTA12/02785 CASA Preston Filtro	#N/A	*C. coli*	Sewage	825	ST-828	1641346	92	Y	Multidrug resistant	SRR8798638
5075	D.40	ZTA12/02785 CPD Preston	2012	*C. coli*	Sewage	1666	ST-828	1869795	151	Y	Non-multidrug resistant	SRR8798637
5076	D.41	ZTA12/02785 CPF Preston	2012	*C. coli*	Sewage	1666	ST-828	1599297	829	Y	Non-multidrug resistant	SRR8798636
5078	D.44	ZTA13/02371 CASA Preston	2013	*C. coli*	Sewage	887	ST-828	1708357	105	Y	Multidrug resistant	SRR8798634
5079	D.45	ZTA13/02372 CASA Filtro	2013	*C. coli*	Sewage	872	ST-828	1846231	80	Y	Multidrug resistant	SRR8798633
5080	D.47	ZTA13/02375 CASA	2013	*C. coli*	Sewage	5659	ST-828	1658188	105	Y	Multidrug resistant	SRR8798671
5081	D.48	ZTA13/02376 CASA Bolton	2013	*C. coli*	Sewage	887	ST-828	1713423	88	Y	Multidrug resistant	SRR8798672
5082	D.49	ZTA13/02377 CASA Preston	2013	*C. coli*	Sewage	825	ST-828	1639550	140	Y	Non-multidrug resistant	SRR8798669
5083	D.50	ZTA13/02378 CASA Preston	2013	*C. coli*	Sewage	872	ST-828	1772422	118	Y	Non-multidrug resistant	SRR8798670
5084	D.52	ZTA13/02380 CASA Preston Filtro	2013	*C. coli*	Sewage	3017	ST-828	1731036	100	Y	Multidrug resistant	SRR8798667
5085	D.53	ZTA13/02381 CASA Preston Filtro	2013	*C. coli*	Sewage	827	ST-828	1755856	256	Y	Non-multidrug resistant	SRR8798668
5086	D.54	ZTA13/02382 CASA	2013	*C. coli*	Sewage	827	ST-828	1672823	148	Y	Non-multidrug resistant	SRR8798665
5088	D.56	ZTA13/02382 CASA Filtro B	2013	*C. coli*	Sewage	854	ST-828	1741608	240	Y	Multidrug resistant	SRR8798676
5089	D.57	ZTA13/02382 CASA Preston Filtro	2013	*C. coli*	Sewage	827	ST-828	1782339	62	Y	Non-multidrug resistant	SRR8798677
5091	D.59	ZTA13/02383 CASA Preston	2013	*C. coli*	Sewage	830	ST-828	1739510	65	Y	Non-multidrug resistant	SRR8798631
5092	D.62	ZTA13/02387 CASA Filtro	2013	*C. coli*	Sewage	827	ST-828	2508613	1189	Y	Non-multidrug resistant	SRR8798651
5094	GR.01	ZTA16/00457	2015	*C. coli*	Human	902	ST-828	1718637	114	Y	Non-multidrug resistant	SRR8798657
5096	GR.03	ZTA16/00460	2015	*C. coli*	Human	827	ST-828	1790885	205	Y	Multidrug resistant	SRR8798659
5097	GR.04	ZTA16/00461	2015	*C. coli*	Human	827	ST-828	1694168	63	Y	Multidrug resistant	SRR8798660
5098	GR.05	ZTA16/00462	2015	*C. coli*	Human		ST-828	1771393	78	Y	Multidrug resistant	SRR8798654
5099	GR.06	ZTA16/00463	2015	*C. coli*	Human	902	ST-828	1648504	58	Y	Non-multidrug resistant	SRR8798655
5101	GR.08	ZTA16/00465	2015	*C. coli*	Human	827	ST-828	1652621	261	Y	Multidrug resistant	SRR8798643
5102	GR.09	ZTA16/00466	2015	*C. coli*	Human	825	ST-828	1637958	185	Y	Non-multidrug resistant	SRR8798646
5103	GR.10	ZTA16/00467	2015	*C. coli*	Human	854	ST-828	1768867	208	Y	Multidrug resistant	SRR8798691
5104	GR.11	ZTA16/00468	2015	*C. coli*	Human	825	ST-828	1677885	126	Y	Non-multidrug resistant	SRR8798673
5105	GR.12	ZTA16/00469	2015	*C. coli*	Human	825	ST-828	1731706	135	Y	Multidrug resistant	SRR8798686
5106	GR.13	ZTA16/00470	2015	*C. coli*	Human	832	ST-828	1646378	98	Y	Non-multidrug resistant	SRR8798685
5107	GR.14	ZTA16/00471	2015	*C. coli*	Human	860	ST-828	1812226	161	Y	Multidrug resistant	SRR8798688
5108	GR.15	ZTA16/00472	2015	*C. coli*	Human	827	ST-828	1786088	106	Y	Multidrug resistant	SRR8798687
5109	GR.16	ZTA16/00474	2015	*C. coli*	Human	5659	ST-828	1619585	220	Y	Non-multidrug resistant	SRR8798682
5110	GR.17	ZTA16/00475	2015	*C. coli*	Human	1055	ST-828	1728298	59	Y	Non-multidrug resistant	SRR8798681
5111	GR.18	ZTA16/00476	#N/A	*C. coli*	Human	3020	ST-828	1706195	88	Y	nd	SRR8798684
5112	GR.19	ZTA16/00477	2015	*C. coli*	Human	3020	ST-828	1719782	225	Y	Multidrug resistant	SRR8798679
5113	GR.20	ZTA16/00478	2015	*C. coli*	Human	7622	ST-828	1072032	1084	Y	Non-multidrug resistant	SRR8798680
5114	GR.21	ZTA16/00480	2015	*C. coli*	Human	1556	ST-828	1669627	531	Y	Multidrug resistant	SRR8798698
5115	GR.22	ZTA16/00481	2015	*C. coli*	Human	827	ST-828	1670322	63	Y	Multidrug resistant	SRR8798699
5116	GR.23	ZTA16/00482	2015	*C. coli*	Human	860	ST-828	1634182	100	Y	Non-multidrug resistant	SRR8798692
5117	GR.24	ZTA16/00485	2015	*C. coli*	Human	5659	ST-828	1684340	116	Y	Multidrug resistant	SRR8798694
5118	GR.25	ZTA16/00486	2015	*C. coli*	Human	825	ST-828	1651024	121	Y	Multidrug resistant	SRR8798652
5119	GR.26	ZTA16/00487	2015	*C. coli*	Human	825	ST-828	1658158	80	Y	Multidrug resistant	SRR8798653
5120	GR.27	ZTA16/00488	2015	*C. coli*	Human	827	ST-828	1670286	96	Y	Multidrug resistant	SRR8798696
5122	GR.29	ZTA16/00490	2015	*C. coli*	Human	3017	ST-828	1969158	443	Y	Non-multidrug resistant	SRR8798656
5123	GR.30	ZTA16/00491	2015	*C. coli*	Human		ST-828	1715453	123	Y	Non-multidrug resistant	SRR8798697
5124	ER.01	ZTA09/01326	2009	*C. coli*	Broiler	825	ST-828	1775530	388	Y	Multidrug resistant	SRR8798628
5125	ER.02	VE08/01737	2008	*C. coli*	Broiler	825	ST-828	1668914	145	Y	Multidrug resistant	SRR8798627
5126	ER.03	VE08/01742	2008	*C. coli*	Broiler	2097	ST-828	1797526	146	Y	Multidrug resistant	SRR8798671
5127	ER.04	ZTA11/01873	2011	*C. coli*	Broiler	901	ST-828	1745160	63	Y	Multidrug resistant	SRR8798672
5128	ER.05	ZTA10/02488	2010	*C. coli*	Broiler	894	ST-828	1697765	94	Y	Multidrug resistant	SRR8798669
5129	ER.06	ZTA09/02173	2009	*C. coli*	Pig	894	ST-828	1658391	115	Y	Multidrug resistant	SRR8798670
5130	ER.07	ZTA11/01966	2011	*C. coli*	Broiler	827	ST-828	1661493	145	Y	Multidrug resistant	SRR8798667
5131	ER.08	VE08/03191	2008	*C. coli*	Pig	1112	ST-828	1771386	104	Y	Multidrug resistant	SRR8798668
5132	ER.09	ZTA09/02282	2009	*C. coli*	Pig	2741	ST-828	1740342	61	Y	Multidrug resistant	SRR8798665
5133	ER.10	ZTA09/02553	2009	*C. coli*	Broiler	830	ST-828	1674093	140	Y	Multidrug resistant	SRR8798666
5137	HCC2	ZTA13/00789	2010	*C. coli*	Human	2077	ST-828	1687507	105	Y	Multidrug resistant	SRR8798677
5170	HCC41	ZTA13/00826	2011	*C. coli*	Human	829	ST-828	1704564	302	Y	Non-multidrug resistant	SRR8798679
5171	HCC43	ZTA13/00827	2011	*C. coli*	Human	829	ST-828	1747636	89	Y	Non-multidrug resistant	SRR8798683
5207	HCC86	ZTA13/00866	2012	*C. coli*	Human	827	ST-828	1659481	83	Y	Multidrug resistant	SRR8785428
5231	HSA16	ZTA13/00889	2010	*C. coli*	Human	829	ST-828	1744869	71	Y	Multidrug resistant	SRR8785413
5236	HSA20	ZTA13/00893	2010	*C. coli*	Human	829	ST-828	1747618	92	Y	Non-multidrug resistant	SRR8785443
5261	HSA46	ZTA13/00918	2010	*C. coli*	Human	827	ST-828	1659277	315	Y	Non-multidrug resistant	SRR8785299
5310	BO.19	ZTA10/00749 CPD	2010	*C. coli*	Cattle	827	ST-828	1675080	61	Y	Multidrug resistant	SRR8785350
5049	D.13	ZTA11/03517-3CPF PRESTON	2011	*C. jejuni*	Sewage			1452660	617	Not used	Sensitive	SRR8785303
5055	D.19	ZTA12/02041CASA	2012	*C. jejuni*	Sewage	2109	ST-45	1690468	145	Y	Non-multidrug resistant	SRR8785470
5077	D.43	ZTA13/02371 CASA Filtro	2013	*C. jejuni*	Sewage	2123	ST-362	1628822	83	Y	Non-multidrug resistant	SRR8798635
5087	D.55	ZTA13/02382 CASA	#N/A	*C. jejuni*	Sewage	22	ST-22	1603476	349	Y	nd	SRR8798666
5090	D.58	ZTA13/02382 CPF	2013	*C. jejuni*	Sewage	22	ST-22	1615139	112	Y	Non-multidrug resistant	SRR8798632
5093	D.63	ZTA13/02388 CASA Filtro A	#N/A	*C. jejuni*	Sewage	1707	ST-607	1802766	114	Y	nd	SRR8798649
5095	GR.02	ZTA16/00459	#N/A	*C. jejuni*	Human	531		1723022	194	Y	nd	SRR8798658
5100	GR.07	ZTA16/00464	#N/A	*C. jejuni*	Human	531		1553007	729	Y	nd	SRR8798644

5136	HCC1	ZTA13/00788	2010	*C. jejuni*	Human	51	ST-443	1615507	355	Y	Non-multidrug resistant	SRR8798676
5138	HCC3	ZTA13/00790	2010	*C. jejuni*	Human	464	ST-464	1731184	228	Y	Non-multidrug resistant	SRR8785408
5139	HCC4	ZTA13/00791	2010	*C. jejuni*	Human			1638262	125	Y	Multidrug resistant	SRR8785381
5140	HCC6	ZTA13/00793	2010	*C. jejuni*	Human	572	ST-206	1679385	228	Y	Non-multidrug resistant	SRR8798651
5141	HCC7	ZTA13/00794	2011	*C. jejuni*	Human	572	ST-206	1689897	144	Y	Non-multidrug resistant	SRR8785402
5142	HCC8	ZTA13/00795	2011	*C. jejuni*	Human	354	ST-354	1737665	111	Y	Non-multidrug resistant	SRR8785427
5143	HCC9	ZTA13/00796	2011	*C. jejuni*	Human	523	ST-658	1664230	582	Y	Non-multidrug resistant	SRR8785432
5144	HCC10	ZTA13/00797	2011	*C. jejuni*	Human	19	ST-21	1713000	125	Y	Non-multidrug resistant	SRR8785475
5145	HCC11	ZTA13/00798	2011	*C. jejuni*	Human	607	ST-607	1748360	171	Y	Non-multidrug resistant	SRR8785476
5146	HCC13	ZTA13/00799	2011	*C. jejuni*	Human	356	ST-353	1715306	255	Y	Non-multidrug resistant	SRR8798678
5147	HCC16	ZTA13/00802	2011	*C. jejuni*	Human	2112	ST-206	1675010	64	Y	Non-multidrug resistant	SRR8798675
5148	HCC17	ZTA13/00803	2011	*C. jejuni*	Human	1044	ST-658	1608142	98	Y	Non-multidrug resistant	SRR8798661
5149	HCC18	ZTA13/00804	2011	*C. jejuni*	Human	47	ST-21	1610319	142	Y	Non-multidrug resistant	SRR8798662
5150	HCC19	ZTA13/00805	2011	*C. jejuni*	Human	464	ST-464	1694653	120	Y	Non-multidrug resistant	SRR8798663
5151	HCC20	ZTA13/00806	2011	*C. jejuni*	Human	859	ST-443	1672281	72	Y	Non-multidrug resistant	SRR8798664
5152	HCC21	ZTA13/00807	2011	*C. jejuni*	Human	50	ST-21	1597814	159	Y	Non-multidrug resistant	SRR8798657
5153	HCC22	ZTA13/00808	2011	*C. jejuni*	Human	21	ST-21	1700885	286	Y	Non-multidrug resistant	SRR8798658
5154	HCC23	ZTA13/00809	2011	*C. jejuni*	Human	1707	ST-607	1788294	152	Y	Non-multidrug resistant	SRR8798659
5155	HCC24	ZTA13/00810	2011	*C. jejuni*	Human	572	ST-206	1643514	363	Y	Non-multidrug resistant	SRR8798660
5156	HCC25	ZTA13/00811	2011	*C. jejuni*	Human	464	ST-464	1725903	240	Y	Non-multidrug resistant	SRR8798654
5157	HCC26	ZTA13/00812	2011	*C. jejuni*	Human	50	ST-21	1684631	251	Y	Multidrug resistant	SRR8798655
5158	HCC27	ZTA13/00813	2011	*C. jejuni*	Human	122	ST-206	1660040	356	Y	Multidrug resistant	SRR8785410
5159	HCC29	ZTA13/00815	2011	*C. jejuni*	Human	2123	ST-362	1592258	129	Y	Non-multidrug resistant	SRR8785411
5160	HCC30	ZTA13/00816	2011	*C. jejuni*	Human	46	ST-206	1583422	789	Y	Multidrug resistant	SRR8785409
5161	HCC31	ZTA13/00817	2011	*C. jejuni*	Human	51	ST-443	1660465	57	Y	Non-multidrug resistant	SRR8798691
5162	HCC32	ZTA13/00818	2011	*C. jejuni*	Human	2844	ST-460	1767716	207	Y	Multidrug resistant	SRR8798673
5163	HCC33	ZTA13/00819	2011	*C. jejuni*	Human	21	ST-21	1730841	113	Y	Non-multidrug resistant	SRR8798693
5164	HCC34	ZTA13/00820	2011	*C. jejuni*	Human	469	ST-42	1618124	186	Y	Multidrug resistant	SRR8785446
5165	HCC35	ZTA13/00821	2011	*C. jejuni*	Human	51	ST-443	1653040	139	Y	Sensitive	SRR8785445
5166	HCC36	ZTA13/00822	2011	*C. jejuni*	Human	53	ST-21	1649932	88	Y	Non-multidrug resistant	SRR8798674
5167	HCC37	ZTA13/00823	2011	*C. jejuni*	Human	441	Unknown	1428187	724	Y	Non-multidrug resistant	SRR8798650
5168	HCC38	ZTA13/00824	2011	*C. jejuni*	Human	523	ST-658	1773054	414	Y	Non-multidrug resistant	SRR8798689
5169	HCC40	ZTA13/00825	2011	*C. jejuni*	Human	21	ST-21	1724122	173	Y	Multidrug resistant	SRR8798690
5172	HCC44	ZTA13/00828	2011	*C. jejuni*	Human	538	ST-45	1550918	460	Y	Non-multidrug resistant	SRR8798695
5173	HCC45	ZTA13/00829	2011	*C. jejuni*	Human	122	ST-206	1686207	58	Y	Non-multidrug resistant	SRR8798699
5174	HCC46	ZTA13/00830	2011	*C. jejuni*	Human	3769	ST-21	1637478	163	Y	Non-multidrug resistant	SRR8798692
5175	HCC47	ZTA13/00831	2011	*C. jejuni*	Human	572	ST-206	1697195	70	Y	Non-multidrug resistant	SRR8798694
5176	HCC48	ZTA13/00832	2011	*C. jejuni*	Human	122	ST-206	1648860	257	Y	Non-multidrug resistant	SRR8798652
5177	HCC49	ZTA13/00833	2011	*C. jejuni*	Human	572	ST-206	1676011	210	Y	Non-multidrug resistant	SRR8798653
5178	HCC50	ZTA13/00834	2011	*C. jejuni*	Human	2180	ST-658	1705752	113	Y	Non-multidrug resistant	SRR8798696
5180	HCC52	ZTA13/00836	2011	*C. jejuni*	Human	5	ST-353	1694793	374	Y	Non-multidrug resistant	SRR8798656
5181	HCC54	ZTA13/00837	2011	*C. jejuni*	Human	48	ST-48	1661946	241	Y	Non-multidrug resistant	SRR8798697
5182	HCC55	ZTA13/00838	2011	*C. jejuni*	Human	443	ST-443	1653389	474	Y	Non-multidrug resistant	SRR8785318
5183	HCC57	ZTA13/00840	2012	*C. jejuni*	Human	22	ST-22	1459780	644	Y	Non-multidrug resistant	SRR8785319
5184	HCC58	ZTA13/00841	2012	*C. jejuni*	Human	464	ST-464	1633957	562	Y	Non-multidrug resistant	SRR8785320
5185	HCC59	ZTA13/00842	2012	*C. jejuni*	Human	354	ST-354	1702806	161	Y	Non-multidrug resistant	SRR8785321
5186	HCC60	ZTA13/00843	2012	*C. jejuni*	Human	772	ST-443	1712599	122	Y	Non-multidrug resistant	SRR8785377
5187	HCC61	ZTA13/00844	2012	*C. jejuni*	Human	50	ST-21	1649532	91	Y	Non-multidrug resistant	SRR8785376
5188	HCC62	ZTA13/00845	2012	*C. jejuni*	Human	2928	ST-443	1629775	90	Y	Non-multidrug resistant	SRR8785375
5189	HCC63	ZTA13/00846	2012	*C. jejuni*	Human	122	ST-206	1718308	324	Y	Non-multidrug resistant	SRR8785374
5190	HCC64	ZTA13/00847	2012	*C. jejuni*	Human	1044	ST-658	1650205	65	Y	Non-multidrug resistant	SRR8785373
5191	HCC65	ZTA13/00848	2012	*C. jejuni*	Human	53	ST-21	1162818	1008	Y	Non-multidrug resistant	SRR8785372
5192	HCC66	ZTA13/00849	2012	*C. jejuni*	Human	450	ST-446	1716080	193	Y	Non-multidrug resistant	SRR8785371
5193	HCC67	ZTA13/00850	2012	*C. jejuni*	Human	53	ST-21	1674047	212	Y	Non-multidrug resistant	SRR8785370
5194	HCC68	ZTA13/00851	2012	*C. jejuni*	Human	50	ST-21	1655957	168	Y	Non-multidrug resistant	SRR8785369
5195	HCC69	ZTA13/00852	2012	*C. jejuni*	Human	22	ST-22	1643811	136	Y	Non-multidrug resistant	SRR8785368
5196	HCC70	ZTA13/00853	2012	*C. jejuni*	Human	3769	ST-21	1467773	681	Y	Non-multidrug resistant	SRR8785403
5197	HCC71	ZTA13/00854	2012	*C. jejuni*	Human	49	ST-49	1604637	130	Y	Non-multidrug resistant	SRR8785400
5198	HCC72	ZTA13/00855	2012	*C. jejuni*	Human	21	ST-21	1670550	55	Y	Non-multidrug resistant	SRR8785401
5199	HCC73	ZTA13/00856	2012	*C. jejuni*	Human	2133		1637551	105	Y	Non-multidrug resistant	SRR8785406
5200	HCC74	ZTA13/00857	2012	*C. jejuni*	Human	356	ST-353	1707470	93	Y	Non-multidrug resistant	SRR8785407
5201	HCC75	ZTA13/00858	2012	*C. jejuni*	Human	2123	ST-362	1588165	93	Y	Non-multidrug resistant	SRR8785404
5202	HCC76	ZTA13/00859	2012	*C. jejuni*	Human	21	ST-21	1652261	173	Y	Non-multidrug resistant	SRR8785405
5203	HCC77	ZTA13/00860	2012	*C. jejuni*	Human	2324		1595036	651	Y	Non-multidrug resistant	SRR8785398
5204	HCC79	ZTA13/00861	2012	*C. jejuni*	Human	583	ST-45	1533839	448	Y	Non-multidrug resistant	SRR8785399
5205	HCC83	ZTA13/00864	2012	*C. jejuni*	Human	354	ST-354	1612158	582	Y	Non-multidrug resistant	SRR8785426
5206	HCC84	ZTA13/00865	2012	*C. jejuni*	Human	227	ST-206	1575516	415	Y	Non-multidrug resistant	SRR8785429
5208	HCC87	ZTA13/00867	2012	*C. jejuni*	Human	22	ST-22	1610109	74	Y	Non-multidrug resistant	SRR8785431
5209	HCC88	ZTA13/00868	2012	*C. jejuni*	Human	233	ST-45	1624603	43	Y	Non-multidrug resistant	SRR8785430
5210	HCC89	ZTA13/00869	2012	*C. jejuni*	Human	2133		1652820	44	Y	Non-multidrug resistant	SRR8785433
5211	HCC90	ZTA13/00870	2012	*C. jejuni*	Human	233	ST-45	1621106	71	Y	Non-multidrug resistant	SRR8785425
5212	HCC91	ZTA13/00871	2012	*C. jejuni*	Human	457		1737153	116	Y	Non-multidrug resistant	SRR8785424
5213	HCC92	ZTA13/00872	2012	*C. jejuni*	Human	464	ST-464	1687567	112	Y	Non-multidrug resistant	SRR8785420
5215	HCC94		2012	*C. jejuni*	Human	464	ST-464	1697852	222	Y	nd	SRR8785421
5216	HCC95	ZTA13/00873	2012	*C. jejuni*	Human	2133	Unknown	1640831	167	Y	Non-multidrug resistant	SRR8785422
5217	HSA1	ZTA13/00874	2010	*C. jejuni*	Human	21	ST-21	1671026	428	Y	Non-multidrug resistant	SRR8785423
5218	HSA2	ZTA13/00875	2010	*C. jejuni*	Human	21	ST-21	1738692	283	Y	Non-multidrug resistant	SRR8785436
5219	HSA3	ZTA13/00876	2010	*C. jejuni*	Human	356	ST-353	1706650	46	Y	Non-multidrug resistant	SRR8785462
5220	HSA4	ZTA13/00877	2010	*C. jejuni*	Human	356	ST-353	1690324	225	Y	Non-multidrug resistant	SRR8785298
5221	HSA5	ZTA13/00878	2010	*C. jejuni*	Human	257	ST-257	1713755	167	Y	Non-multidrug resistant	SRR8785353
5222	HSA6	ZTA13/00879	2010	*C. jejuni*	Human	2274		1755392	150	Y	Non-multidrug resistant	SRR8785354
5223	HSA7	ZTA13/00880	2010	*C. jejuni*	Human			1689815	566	Y	Non-multidrug resistant	SRR8785355
5224	HSA8	ZTA13/00881	2010	*C. jejuni*	Human	21	ST-21	1696921	82	Y	Non-multidrug resistant	SRR8785356
5225	HSA9	ZTA13/00882	2010	*C. jejuni*	Human	443	ST-443	1714260	101	Y	Non-multidrug resistant	SRR8785357
5226	HSA10	ZTA13/00883	2010	*C. jejuni*	Human	441		1634175	69	Y	Non-multidrug resistant	SRR8785416
5227	HSA11	ZTA13/00884	2010	*C. jejuni*	Human	883	ST-21	1574768	494	Y	Non-multidrug resistant	SRR8785417
5228	HSA12	ZTA13/00885	2010	*C. jejuni*	Human	22	ST-22	1600901	160	Y	Sensitive	SRR8785418
5229	HSA13	ZTA13/00886	2010	*C. jejuni*	Human	990	ST-257	1672162	149	Y	Non-multidrug resistant	SRR8785419
5230	HSA14	ZTA13/00887	2010	*C. jejuni*	Human	22	ST-22	1605633	136	Y	Non-multidrug resistant	SRR8785412
5233	HSA17	ZTA13/00890	2010	*C. jejuni*	Human	5	ST-353	1763615	118	Y	Non-multidrug resistant	SRR8785439
5234	HSA18	ZTA13/00891	2010	*C. jejuni*	Human		ST-443	1617661	146	Y	Sensitive	SRR8785438
5235	HSA19	ZTA13/00892	2010	*C. jejuni*	Human		ST-443	1612471	213	Y	Sensitive	SRR8785437
5237	HSA21	ZTA13/00894	2010	*C. jejuni*	Human	990	ST-257	1714038	139	Y	Non-multidrug resistant	SRR8785442
5238	HSA22	ZTA13/00895	2010	*C. jejuni*	Human	356	ST-353	1754086	38	Y	Non-multidrug resistant	SRR8785441
5239	HSA23	ZTA13/00896	2010	*C. jejuni*	Human	53	ST-21	1133123	1110	Y	Multidrug resistant	SRR8785440
5240	HSA24	ZTA13/00897	2010	*C. jejuni*	Human	572	ST-206	1729006	171	Y	Non-multidrug resistant	SRR8785435
5242	HSA26	ZTA13/00899	2010	*C. jejuni*	Human	572	ST-206	1503284	783	Y	Non-multidrug resistant	SRR8785434
5243	HSA27	ZTA13/00900	2010	*C. jejuni*	Human	990	ST-257	1731259	27	Y	Non-multidrug resistant	SRR8785464
5245	HSA29	ZTA13/00902	2010	*C. jejuni*	Human	305	ST-574	1740194	226	Y	Non-multidrug resistant	SRR8785465
5246	HSA30	ZTA13/00903	2010	*C. jejuni*	Human	48	ST-48	1688756	480	Y	Non-multidrug resistant	SRR8785463
5247	HSA31	ZTA13/00904	2010	*C. jejuni*	Human	440	ST-443	1654089	162	Y	Non-multidrug resistant	SRR8785460
5248	HSA32	ZTA13/00905	2010	*C. jejuni*	Human	441		1613513	167	Y	Multidrug resistant	SRR8785461
5249	HSA33	ZTA13/00906	2010	*C. jejuni*	Human	51	ST-443	1661983	128	Y	Non-multidrug resistant	SRR8785458
5250	HSA35	ZTA13/00907	2010	*C. jejuni*	Human	443	ST-443	1697702	189	Y	Non-multidrug resistant	SRR8785459
5251	HSA36	ZTA13/00908	2010	*C. jejuni*	Human	354	ST-354	1603506	549	Y	Non-multidrug resistant	SRR8785466

5252	HSA37	ZTA13/00909	2010	*C. jejuni*	Human	990	ST-257	1651217	345	Y	Non-multidrug resistant	SRR8785467
5253	HSA38	ZTA13/00910	2010	*C. jejuni*	Human	354	ST-354	1650110	568	Y	Non-multidrug resistant	SRR8785296
5254	HSA39	ZTA13/00911	2010	*C. jejuni*	Human			1187596	938	Y	Non-multidrug resistant	SRR8785295
5255	HSA40	ZTA13/00912	2010	*C. jejuni*	Human	441		1620762	140	Y	Multidrug resistant	SRR8785297
5256	HSA41	ZTA13/00913	2010	*C. jejuni*	Human	356	ST-353	1452912	917	Y	Non-multidrug resistant	SRR8785292
5257	HSA42	ZTA13/00914	2010	*C. jejuni*	Human	464	ST-464	1716511	206	Y	Non-multidrug resistant	SRR8785291
5258	HSA43	ZTA13/00915	2010	*C. jejuni*	Human		ST-354	1734414	225	Y	Non-multidrug resistant	SRR8785294
5259	HSA44	ZTA13/00916	2010	*C. jejuni*	Human	21	ST-21	1629536	524	Y	Non-multidrug resistant	SRR8785293
5260	HSA45	ZTA13/00917	2010	*C. jejuni*	Human	52	ST-52	1714057	342	Y	Non-multidrug resistant	SRR8785300
5262	HSA47	ZTA13/00919	2010	*C. jejuni*	Human	22	ST-22	1585839	301	Y	Non-multidrug resistant	SRR8785351
5263	HSA48	ZTA13/00920	2010	*C. jejuni*	Human	122	ST-206	1589773	375	Y	Non-multidrug resistant	SRR8785352
5264	AV.01	ZTA10/00684 CPD	2010	*C. jejuni*	Broiler	45	ST-45	1632398	157	Y	Non-multidrug resistant	SRR8785338
5267	AV.04	ZTA10/01723 CPD	2010	*C. jejuni*	Broiler	354	ST-354	1677109	214	Y	Multidrug resistant	SRR8785337
5270	AV.07	ZTA10/00766 CPD	2010	*C. jejuni*	Broiler	227	ST-206	1712981	147	Y	Multidrug resistant	SRR8785336
5271	AV.08	ZTA10/01430 CPD	2010	*C. jejuni*	Broiler	4016	ST-42	1608295	441	Y	Non-multidrug resistant	SRR8785335
5272	AV.09	ZTA10/00909 CPD	2010	*C. jejuni*	Broiler	464	ST-464	1734911	184	Y	Multidrug resistant	SRR8785342
5274	AV.11	ZTA10/02105 CPD	2010	*C. jejuni*	Broiler	3030		1643257	68	Y	Multidrug resistant	SRR8785341
5275	AV.12	ZTA10/02531 CPF	2010	*C. jejuni*	Broiler	572	ST-206	1765209	341	Y	Multidrug resistant	SRR8785340
5276	AV.13	ZTA10/02577 CPD	2010	*C. jejuni*	Broiler	137	ST-45	1591527	136	Y	Multidrug resistant	SRR8785339
5279	AV.16	ZTA10/01722 CPD	2010	*C. jejuni*	Broiler	7110	ST-607	1794198	114	Y	Non-multidrug resistant	SRR8785334
5281	AV.18	ZTA10/02640 CPF	2010	*C. jejuni*	Broiler	52	ST-52	1685326	176	Y	Non-multidrug resistant	SRR8785333
5282	AV.19	ZTA10/01246 CPD	2010	*C. jejuni*	Broiler	1710		1694517	198	Y	Non-multidrug resistant	SRR8785391
5284	AV.21	ZTA10/02641 CPD	2010	*C. jejuni*	Broiler	441		1669889	48	Y	Non-multidrug resistant	SRR8785390
5285	AV.22	ZTA11/01072 CP	2011	*C. jejuni*	Broiler	137	ST-45	1587285	159	Y	Multidrug resistant	SRR8785393
5286	AV.23	ZTA11/01302 CP	2011	*C. jejuni*	Broiler	45	ST-45	1655424	304	Y	Multidrug resistant	SRR8785392
5287	AV.24	ZTA11/01874 CP	2011	*C. jejuni*	Broiler	137	ST-45	1587020	161	Y	Multidrug resistant	SRR8785395
5288	AV.25	ZTA11/02059 CP	2010	*C. jejuni*	Broiler	469	ST-42	1592332	307	Y	Non-multidrug resistant	SRR8785394
5289	AV.26	ZTA11/02314 CP	2011	*C. jejuni*	Broiler	21	ST-21	1536243	633	Y	Non-multidrug resistant	SRR8785397
5291	AV.28	ZTA11/03523	2011	*C. jejuni*	Broiler	1044	ST-658	1669660	267	Y	Non-multidrug resistant	SRR8785396
5293	BO.02	ZTA10/01617 CPD	2010	*C. jejuni*	Cattle	4016	ST-42	1610199	250	Y	Non-multidrug resistant	SRR8785389
5295	BO.04	ZTA10/02583 CPF	2010	*C. jejuni*	Cattle	61	ST-61	1648771	132	Y	Non-multidrug resistant	SRR8785388
5296	BO.05	ZTA10/00655 CPD	2010	*C. jejuni*	Cattle	122	ST-206	1686208	77	Y	Non-multidrug resistant	SRR8785364
5297	BO.06	ZTA10/00681 CPD	2010	*C. jejuni*	Cattle	354	ST-354	1712074	181	Y	Non-multidrug resistant	SRR8785365
5298	BO.07	ZTA10/00761 CPD	2010	*C. jejuni*	Cattle	464	ST-464	1727851	169	Y	Non-multidrug resistant	SRR8785366
5299	BO.08	ZTA10/01043 CPD	2010	*C. jejuni*	Cattle	50	ST-21	1677598	160	Y	Non-multidrug resistant	SRR8785367
5300	BO.09	ZTA10/01185 CPD	2010	*C. jejuni*	Cattle	61	ST-61	1656093	62	Y	Multidrug resistant	SRR8785360
5301	BO.10	ZTA10/01678 CPD	2010	*C. jejuni*	Cattle	21	ST-21	1719251	246	Y	Multidrug resistant	SRR8785361
5302	BO.11	ZTA10/00700 CPD	2010	*C. jejuni*	Cattle	42	ST-42	1564065	276	Y	Sensitive	SRR8785362
5303	BO.12	ZTA10/00900 CPF	2010	*C. jejuni*	Cattle	19	ST-21	1656504	80	Y	Non-multidrug resistant	SRR8785363
5304	BO.13	ZTA10/00951 CPD	2010	*C. jejuni*	Cattle	42	ST-42	1594226	51	Y	Multidrug resistant	SRR8785358
5305	BO.14	ZTA10/01185 CPD	2010	*C. jejuni*	Cattle	61	ST-61	1647767	138	Y	Multidrug resistant	SRR8785359
5306	BO.15	ZTA10/01333 CPD	2010	*C. jejuni*	Cattle	441		1622297	117	Y	Non-multidrug resistant	SRR8785346
5307	BO.16	ZTA10/01411 CPD	2010	*C. jejuni*	Cattle	61	ST-61	1757133	121	Y	Non-multidrug resistant	SRR8785345
5308	BO.17	ZTA10/02289 CPD	2010	*C. jejuni*	Cattle	122	ST-206	1756195	45	Y	Multidrug resistant	SRR8785344
5309	BO.18	ZTA10/02492 CPD	2010	*C. jejuni*	Cattle	575	ST-42	1699842	87	Y	Sensitive	SRR8785343
5311	BO.20	ZTA10/00575 CPD	2010	*C. jejuni*	Cattle	21	ST-21	1713503	136	Y	Multidrug resistant	SRR8785349
5313	BO.22	ZTA11/00934 CP	2011	*C. jejuni*	Cattle	21	ST-21	1714986	96	Y	Multidrug resistant	SRR8785348
5316	BO.25	ZTA11/03537 CP	2011	*C. jejuni*	Cattle	61	ST-61	1595849	493	Y	Non-multidrug resistant	SRR8785347
5317	BO.26	ZTA11/03821 CP	2011	*C. jejuni*	Cattle	21	ST-21	1708914	102	Y	Non-multidrug resistant	SRR8785312
5318	BO.27	ZTA11/02705 CP	2011	*C. jejuni*	Cattle	61	ST-61	1578679	687	Y	Non-multidrug resistant	SRR8785301
5321	BO.30	ZTA11/01052 CP	2011	*C. jejuni*	Cattle	61	ST-61	1634301	318	Y	Non-multidrug resistant	SRR8785329
5322	BO.31	ZTA11/03823 CP	2011	*C. jejuni*	Cattle	61	ST-61	1645542	193	Y	Non-multidrug resistant	SRR8785330
5324	BO.33	ZTA11/02069 CP	2011	*C. jejuni*	Cattle	52	ST-52	1731269	61	Y	Multidrug resistant	SRR8785327
5325	BO.34	ZTA11/01530 CP	2011	*C. jejuni*	Cattle	586		1689073	88	Y	Non-multidrug resistant	SRR8785328
5327	BO.36	ZTA11/01842 CP	2011	*C. jejuni*	Cattle	42	ST-42	1743650	74	Y	Non-multidrug resistant	SRR8785325
