# Supplementary material for: Gene pool transmission of multidrug resistance among Campylobacter from livestock, sewage and human disease
Source: Environ Microbiol. 2019 Aug 27;21(12):4597–613. doi: 10.1111/1462-2920.14760 (PMC6916351; doi:10.1111/1462-2920.14760)
Supplement: Supplementary file 2 — Table S2. Isolates and their MIC against different antibiotics used in this study. [file EMI-21-4597-s004.pdf]

Table S2. Isolates and their MIC against different antibiotics used in this study

Antibiotic	Species	MIC Range (mg/L)	ECOFF (mg/L)*	Isolates per MIC value (mg/L)												Resistant isolates (%)
				0.12	0.25	0.5	1	2	4	8	16	32	64	128	>128	
Ciprofloxacin	*C. jejuni*	0.12 - 16	0.5	8	6	2	6	1		42	22	46	24	2	3	146/163 (89.57%)
	C. coli		0.5	1	3	1		1	17	57	7		2	2		86/91 (94.50%)
Nalidixic acid	*C. jejuni*	1 - 64	16				3		3	14		2	9	72	33	116/163 (71.16%)
	C. coli		16							4	2	14	14	56		84/91 (92.31%)
Tetracycline	*C. jejuni*	0.5 - 64	1		7	1	7	5	11	1	4	43	26	42	17	149/163 (91.41%)
	C. coli		2			2	2	1				58	12	14	2	86/91 (94.5%)
Erythromycin	*C. jejuni*	1 - 128	4	1	1	48	66	40	3	1			3			4/163 (2.45%)
	C. coli		8			2	45	19	1	1	1	2	8	7	5	23/91 (25.3%)
Streptomycin	*C. jejuni*	0.25 - 16	4				27	57	54	7	1	13		1	2	24/163 (14.72%)
	C. coli		4		1	7	9	10	6	3	3	43	7	2		58/91 (63.7%)
Gentamycin	*C. jejuni*	0.12 - 16	2		4	10	51	96	1		1					2/163 (1.23%)
	C. coli		2	12	21	34	6	8				10				10/91 (11%)

*EUCAST: *C. coli* and *C. jejuni* data from EUCAST (last accessed on: June 2017)
