# Supplementary material for: Gene pool transmission of multidrug resistance among Campylobacter from livestock, sewage and human disease
Source: Environ Microbiol. 2019 Aug 27;21(12):4597–613. doi: 10.1111/1462-2920.14760 (PMC6916351; doi:10.1111/1462-2920.14760)
Supplement: Supplementary file 3 — Table S3. Resistance phenotype–genotype correlations among Campylobacter isolates. [file EMI-21-4597-s005.pdf]

Table S3. Resistance phenotype-genotype correlations among *Campylobacter* isolates

Drug class	Antibiotic tested	Phenotypes (no. of isolates)[a]	Genotypes (no. of isolates)	Correlation %[b]	p-value[d]
Tetracyclines	Tetracycline	R, 235	*tetO* (n=194)	82.3 (194/235)	<0.001
		S, 20	None	100 (20/20)	
Macrolides	Erythromycin	R, 27	*ermB* (n=1)	3.7 (1/27)[c]	0.11
		S, 227	None	100 (227/227)	
Aminoglycosides	Streptomycin	R, 82	*ant-like* B (n=6), *ant-like* A (n=34), *sat-1* (n=3), *aad9* (n=18), *ant(6)-Ib* (n=23), *ant(6)-Ia* (n=14), *sat-4* (n=14), *aph(3)-IIIa* (n=5), *hpt* (n=9), *aph(2)_IIIa* (n=7), *apmA* (n=2)	69.5 (57/82)	<0.001
		S, 172	*ant-like* B (n=5), *ant-like* A (n=5), *ant(6)-Ia* (n=2), *sat-4* (n=2), *aph(3)-IIIa* (n=2)	4.1 (7/172)	
	Gentamicin	R, 12	*ant-like* B (n=1), *ant-like* A (n=3), *aad9* (n=9), *ant(6)-Ib* (n=9), *ant(6)-Ia* (n=6), *sat-4* (n=7), *hpt* (n=6), *aph(2)-IIIa* (n=7), *apmA* (n=2)	91.7 (11/12)	<0.001
		S, 242	*ant-like* B (n=10), *ant-like* A (n=36), *sat-1* (n=3), *aad9* (n=9), *ant(6)-Ib* (n=14), *ant(6)-Ia* (n=10), *sat-4* (n=9), *aph(3)-IIIa* (n=7), *hpt* (n=3)	21.9 (53/242)	

[a] *R, resistant; S, susceptible*

[b] *Number of isolates carrying an AMR gene confering resistance to the associated antibiotic/number of isolates with the resistant phenotype*

[c] *A number of 27 isolates were resistant to erythromycin, but only one isolate contained the corresponding resistant gene*

[d] *Fisher's exact test with 95% confidence interval*
