# Supplementary material for: Gene pool transmission of multidrug resistance among Campylobacter from livestock, sewage and human disease
Source: Environ Microbiol. 2019 Aug 27;21(12):4597–613. doi: 10.1111/1462-2920.14760 (PMC6916351; doi:10.1111/1462-2920.14760)
Supplement: Supplementary file 4 — Table S4. Antibiotic drug classes: mechanism of action/resistance and AMR genes. [file EMI-21-4597-s006.pdf]

Table S4. Antibiotic drug classes, their mechanism of action, their associated AMR genes and their resistance mechanisms

Drug class	Antibiotics	Antibiotic Mechanism	Antimicrobial genes	Resistance Mechanism
Fluoroquinolones	Ciprofloxacin/nalidixic acid	Target DNA gyrase and topoisomerase IV, impair DNA replication	-	Point mutations in GyrA gene, functional efflux pump (CmeABC)
Tetracyclines	Tetracycline	Protein synthesis inhibition - prevent attachment of aminoacyl-tRNA to the ribosomal acceptor site	*tet* O	Ribosomal protection proteins actively removing tetracycline from the ribosome in a GTP hydrolysis-dependent fashion
Macrolides	Erythromycin, Azithromycin	Target 50S subunit of bacterial ribosome and inhibit protein synthesis through interference with the peptide translocation step	*ermB*	Modifications of the ribosomal target via enzyme-mediated methylation, point mutation in domain V of the 23S rRNA and/or ribosomal proteins L4 and L22, functional multidrug efflux pump (CmeABC)
Aminoglycosides	Apramycin, Gentamicin, Streptomycin, Kanamycin, Hygromycin B	Bactericidal action - bind to ribosomes and inhibits protein synthesis	*aph(2)-IIIa, aph(3)-IIIa, sat-1, sat-4, aad9, ant-like* B, *ant-like* A, *ant(6)-Ia, ant(6)-Ib, hpt, apmA*	Modification of the antibiotic structure - Phosphorylation of the 3' hydroxyl group of aminoglycosides (e.g. aminoglycoside acetyltransferases, phosphotransferases and nucleotidyl-transferases)
Phenicols	Chloramphenicol	Bacteriostatic action - binds to bacterial ribosome via blocking the peptidyl transferase and inhibits protein synthesis	-	Enzymatic inactivation by chloramphenicol acetyltransferases, target site mutation in 23S rRNA, target site modification in 23S rRNA by rRNA methytransferase, enhanced extrusion by efflux pumps
Lincosamides	Lincomycin, Clindamycin	Protein synthesis inhibition - prevent peptidyltransferase binding to the 50S subunit of the bacterial ribosome	*lnuC*	Phosphorylation and nucleotidylation of the hydroxyl group of lincosamides by lincosamide nucleotidyltransferases, N^6 dimethylation of an adenine residue of the 23S rRNA molecule
β-lactams	Amoxicillin, Ampicillin	Inhibit bacterial cell wall biosynthesis	*bla* OXA-61	enzymatic inactivation, reduced uptake, functional efflux pumps (CmeABC)
