# Supplementary material for: Gene pool transmission of multidrug resistance among Campylobacter from livestock, sewage and human disease
Source: Environ Microbiol. 2019 Aug 27;21(12):4597–613. doi: 10.1111/1462-2920.14760 (PMC6916351; doi:10.1111/1462-2920.14760)
Supplement: Supplementary file 5 — Table S5. Genomic and phenotypic details of all isolates used in this study. [file EMI-21-4597-s007.pdf]

Table S5. Genomic and phenotypic details of all isolates used in this study

Id	Strain name	Species	Nalidixic acid (>16)	Ciprofloxacin (>0.5)	Tetracycline (>2)	Erythromycin (>8)	Streptomycin (>4)	Gentamycin (>2)	*bla* OXA-61	*tet* O	*ant-like* B	*ant-like* A	*ant(6)-Ia*	*sat-4*	*lnuC*	*ant(6)-Ib*	*aad9*	*aph(3)-IIIa*	*aph(2)-IIIa*	*hpt*	*apmA*	*sat-1*	*ermB*
5040	D.01	*C. coli*	128	8	32	1	32	0.5	0	2	0	0	0	0	0	2	2	0	0	2	0	0	0
5041	D.03	*C. coli*	128	8	32	2	32	0.5	0	3	0	2	0	0	0	0	0	0	0	0	0	0	0
5042	D.06	*C. coli*	128	8	32	1	32	0.5	0	4	0	3	0	0	0	0	0	0	0	0	0	0	0
5043	D.07	*C. coli*	128	8	32	1	32	32	0	0	0	0	2	2	0	2	3	0	2	1	0	0	0
5044	D.08	*C. coli*	128	8	32	1	32	0.5	0	4	0	0	0	0	0	0	0	0	0	0	0	0	0
5045	D.09	*C. coli*	8	0.5	32	1	32	0.5	0	4	0	4	0	0	0	0	0	0	0	0	0	0	0
5046	D.10	*C. coli*	128	8	32	1	2	0.5	0	5	0	0	0	0	0	0	0	0	0	0	0	0	0
5047	D.11	*C. coli*	128	8	1	0.5	2	0.25	0	0	0	0	0	0	0	0	0	0	0	0	0	0	0
5048	D.12	*C. coli*	128	8	32	64	2	0.5	0	6	0	0	0	0	0	0	0	0	0	0	0	0	0
5049	D.13	*C. jejuni*	4	0.12	0.25	0.5	2	0.5	0	0	0	0	0	0	0	0	0	0	0	0	0	0	0
5050	D.14	*C. coli*	8	0.12	32	1	32	0.5	1	4	0	3	0	0	0	0	0	0	0	0	0	0	0
5051	D.15	*C. coli*	128	8	32	1	32	0.5	0	4	0	0	0	0	0	0	0	0	0	0	0	0	0
5052	D.16	*C. coli*	128	8	32	64	32	0.25	0	7	0	0	0	0	0	0	0	0	0	0	0	0	0
5053	D.17	*C. coli*	128	8	32	32	32	0.5	0	7	0	3	0	0	0	0	0	0	0	0	0	0	0
5054	D.18	*C. coli*	128	8	32	32	32	0.5	0	7	0	5	0	0	0	0	0	0	0	0	0	0	0
5055	D.19	*C. jejuni*	64	8	0.25	0.5	1	0.25	0	0	0	0	0	0	0	0	0	0	0	0	0	0	0
5056	D.20	*C. coli*	128	8	32	2	4	0.5	2	8	0	0	0	0	2	0	0	0	0	0	0	0	0
5057	D.21	*C. coli*	128	8	32	2	32	0.5	0	4	0	5	0	0	0	0	0	0	0	0	0	0	0
5058	D.22	*C. coli*	128	8	32	1	2	1	2	8	0	0	0	0	2	0	0	0	0	0	0	0	0
5059	D.23	*C. coli*	128	8	32	1	32	0.5	0	4	0	3	0	0	0	0	0	0	0	0	0	0	0
5060	D.24	*C. coli*	128	8	32	2	32	0.5	4	4	0	5	0	0	0	0	0	0	0	0	0	0	0
5061	D.25	*C. coli*	128	8	32	2	32	0.5	0	4	0	0	0	0	0	0	0	0	0	0	0	0	0
5062	D.26	*C. coli*	128	8	32	2	2	0.5	4	8	0	0	0	0	2	0	0	0	0	0	0	0	0
5063	D.27	*C. coli*	128	8	32	2	4	0.5	0	0	0	0	0	0	2	0	0	0	0	0	0	0	0
5064	D.28	*C. coli*	8	0.25	32	2	32	0.5	1	9	0	5	0	0	0	0	0	0	0	0	0	0	0
5065	D.29	*C. coli*	128	8	32	2	32	0.5	5	10	0	5	0	0	0	0	0	0	0	0	0	0	0
5066	D.31	*C. coli*	128	8	32	1	32	32	6	0	0	0	3	3	2	3	4	0	1	1	0	0	0
5067	D.32	*C. coli*	16	0.25	32	2	32	0.5	1	11	0	5	0	0	0	0	0	0	0	0	0	0	0
5068	D.33	*C. coli*	128	8	32	4	2	0.5	7	4	0	0	0	0	0	0	0	0	0	0	0	0	0
5069	D.34	*C. coli*	128	8	32	2	32	32	5	0	0	0	4	3	3	3	3	0	1	1	0	0	0
5070	D.35	*C. coli*	128	8	32	2	32	32	0	0	0	0	5	3	3	3	3	0	1	1	0	0	0
5072	D.37	*C. coli*	128	8	32	2	32	0.5	1	4	2	6	0	0	0	2	3	0	0	0	0	0	0
5073	D.38	*C. coli*	128	8	32	1	32	0.25	0	4	3	7	0	0	0	2	5	0	0	0	0	0	0
5074	D.39	*C. coli*	128	8	32	2	32	0.5	0	4	0	5	0	0	0	0	0	0	0	0	0	0	0
5075	D.40	*C. coli*	128	8	32	1	2	0.25	8	4	0	0	0	0	0	0	0	0	0	0	0	0	0
5076	D.41	*C. coli*	128	8	32	1	2	0.25	0	4	0	0	0	0	0	0	0	0	0	0	0	0	0
5077	D.43	*C. jejuni*	128	8	32	0.5	1	0.25	0	12	0	0	0	0	0	0	0	0	0	0	0	0	0
5078	D.44	*C. coli*	128	8	32	1	8	0.5	5	13	0	0	0	0	0	0	0	0	0	0	0	0	0
5079	D.45	*C. coli*	128	8	32	64	32	0.5	1	14	0	0	6	0	0	0	0	0	0	0	0	0	0
5080	D.47	*C. coli*	128	8	32	1	32	0.5	0	4	0	0	0	0	0	2	3	0	0	0	0	0	0
5081	D.48	*C. coli*	128	8	32	1	16	0.25	5	13	0	0	0	0	0	0	0	0	0	0	0	0	0
5082	D.49	*C. coli*	128	8	2	0.5	32	0.5	0	0	0	5	0	0	0	0	0	0	0	0	0	0	0
5083	D.50	*C. coli*	128	8	32	1	2	0.5	1	15	0	0	0	0	0	0	0	0	0	0	0	0	0
5084	D.52	*C. coli*	128	8	32	64	1	0.25	8	4	0	0	0	0	0	0	0	0	0	0	0	0	0
5085	D.53	*C. coli*	128	8	32	1	1	0.5	9	16	4	8	0	0	0	0	0	0	0	0	0	0	0
5086	D.54	*C. coli*	128	8	1	1	2	0.5	0	0	0	0	0	0	0	0	0	0	0	0	0	0	0
5087	D.55	*C. jejuni*	128	8	1	NT	NT	NT	0	0	0	0	0	0	0	0	0	0	0	0	0	0	0
5088	D.56	*C. coli*	128	8	32	64	32	0.5	0	17	0	0	7	4	0	0	0	1	0	1	0	0	0
5089	D.57	*C. coli*	128	8	32	1	1	0.25	1	16	2	6	0	0	0	0	0	0	0	0	0	0	0
5090	D.58	*C. jejuni*	128	8	32	1	1	0.25	5	0	0	0	0	0	0	0	0	0	0	0	0	0	0
5091	D.59	*C. coli*	64	8	32	2	1	0.25	8	4	0	0	0	0	0	0	0	0	0	0	0	0	0
5092	D.62	*C. coli*	128	8	32	2	1	0.5	1	16	2	6	0	0	0	0	0	0	0	0	0	0	0
5093	D.63	*C. jejuni*	NT	NT	NT	NT	NT	NT	0	18	0	0	0	0	0	0	0	0	0	0	0	0	0
5094	GR.01	*C. coli*	64	4	0.5	1	0.5	0.25	5	0	0	0	0	0	0	0	0	0	0	0	0	0	0
5095	GR.02	*C. jejuni*	NT	NT	NT	NT	NT	NT	10	19	0	0	0	0	0	0	0	0	0	0	0	0	0
5096	GR.03	*C. coli*	32	4	128	1	32	0.12	1	20	0	5	0	0	0	0	0	0	0	0	0	0	0
5097	GR.04	*C. coli*	32	4	64	1	32	0.25	1	16	5	9	0	0	0	0	0	0	0	0	0	0	0
5098	GR.05	*C. coli*	64	4	32	>128	16	0.12	11	21	0	3	7	4	0	0	0	1	0	1	0	0	0
5099	GR.06	*C. coli*	64	4	0.5	1	0.5	0.12	5	0	0	0	0	0	0	0	0	0	0	0	0	0	0
5100	GR.07	*C. jejuni*	NT	NT	NT	NT	NT	NT	0	17	0	0	0	0	0	0	0	0	0	0	0	0	0
5101	GR.08	*C. coli*	32	4	128	1	32	0.12	0	16	0	5	0	0	0	0	0	0	0	0	0	0	0
5102	GR.09	*C. coli*	64	4	128	1	1	0.25	5	4	0	0	0	0	0	0	0	0	0	0	0	0	0
5103	GR.10	*C. coli*	64	8	128	>128	32	32	0	0	0	3	2	4	0	0	4	0	0	1	0	0	0
5104	GR.11	*C. coli*	64	4	128	1	1	0.25	5	4	0	0	0	0	0	0	0	0	0	0	0	0	0
5105	GR.12	*C. coli*	64	8	64	>128	32	0.25	0	4	0	3	0	0	0	0	0	0	0	0	0	0	0
5106	GR.13	*C. coli*	32	16	64	1	0.5	0.12	0	22	0	0	0	0	0	0	0	0	0	0	0	0	0
5107	GR.14	*C. coli*	32	4	32	128	0.5	0.12	0	4	0	0	0	0	0	0	0	0	0	0	0	0	0
5108	GR.15	*C. coli*	32	16	128	1	32	32	1	0	2	6	0	0	0	2	6	0	1	0	0	0	0
5109	GR.16	*C. coli*	32	4	128	1	0.5	0.25	5	6	0	0	0	0	0	0	0	0	0	0	0	0	0
5110	GR.17	*C. coli*	64	4	128	1	0.5	0.25	0	4	0	0	0	0	0	0	0	0	0	0	0	0	0
5111	GR.18	*C. coli*	NT	NT	NT	NT	NT	NT	0	4	1	0	0	5	0	0	0	2	0	0	0	0	0
5112	GR.19	*C. coli*	32	8	128	1	32	0.12	0	23	0	0	8	5	0	0	0	2	0	0	0	0	0
5113	GR.20	*C. coli*	64	4	64	1	1	0.25	0	4	0	0	0	0	0	0	0	0	0	0	0	0	0
5114	GR.21	*C. coli*	32	2	64	1	32	0.12	0	24	0	0	0	0	0	2	4	0	0	0	0	0	0
5115	GR.22	*C. coli*	32	4	128	1	32	0.25	1	4	0	3	0	0	0	0	0	0	0	0	0	0	0
5116	GR.23	*C. coli*	64	4	64	1	0.5	0.12	5	4	0	0	0	0	0	0	0	0	0	0	0	0	0
5117	GR.24	*C. coli*	64	16	128	>128	32	0.25	5	6	0	0	0	0	0	2	4	0	0	0	0	0	0
5118	GR.25	*C. coli*	64	8	128	1	16	0.12	0	4	0	0	0	0	0	0	0	0	0	0	0	0	0
5119	GR.26	*C. coli*	128	8	64	>128	8	0.25	0	4	0	3	0	0	0	0	0	0	0	0	0	0	0
5120	GR.27	*C. coli*	64	8	64	1	32	0.25	1	16	2	6	0	0	0	0	0	0	0	0	0	0	0
5122	GR.29	*C. coli*	32	4	64	1	0.25	0.12	8	0	0	0	0	0	0	0	0	0	0	0	0	0	0
5123	GR.30	*C. coli*	32	4	64	1	1	0.12	6	4	8	0	0	4	2	0	0	1	0	0	0	0	0
5124	ER.01	*C. coli*	128	8	32	128	64	1	0	4	0	0	0	0	0	0	0	0	0	0	0	0	0
5125	ER.02	*C. coli*	128	8	32	128	64	32	5	0	0	0	2	2	0	2	4	0	1	1	0	0	0
5126	ER.03	*C. coli*	128	16	32	128	64	32	2	0	0	0	0	3	0	2	0	0	1	0	0	0	0
5127	ER.04	*C. coli*	32	8	32	64	32	0.5	5	25	0	0	0	0	0	2	3	0	0	0	0	0	0
5128	ER.05	*C. coli*	128	8	32	64	32	32	5	0	0	0	0	0	0	2	4	0	0	0	1	0	2
5129	ER.06	*C. coli*	128	4	32	16	64	1	0	26	0	1	0	0	1	4	4	0	0	0	0	0	0
5130	ER.07	*C. coli*	128	8	32	64	32	2	0	4	0	5	0	0	0	0	0	0	0	0	0	0	0
5131	ER.08	*C. coli*	128	8	32	128	64	2	5	4	0	3	0	0	0	0	0	0	0	0	0	0	0
5132	ER.09	*C. coli*	128	8	32	128	64	32	5	27	0	3	0	0	0	0	0	0	0	0	0	0	0
5133	ER.10	*C. coli*	128	16	32	128	4	1	8	4	0	0	0	0	0	0	0	0	0	0	0	0	0
5136	HCC1	*C. jejuni*	128	32	32	1	2	1	12	28	0	0	0	0	0	0	0	0	0	0	0	0	0
5137	HCC2	*C. coli*	128	64	128	1	128	2	13	4	0	3	0	0	0	2	3	0	0	0	0	0	0
5138	HCC3	*C. jejuni*	128	32	128	0.25	2	1	5	17	0	0	0	0	0	0	0	0	0	0	0	0	0
5139	HCC4	*C. jejuni*	128	64	>512	1	32	16	12	0	0	0	0	0	0	2	4	1	0	0	1	0	0
5140	HCC6	*C. jejuni*	128	32	128	1	4	2	14	4	0	0	0	0	0	0	0	0	0	0	0	0	0
5141	HCC7	*C. jejuni*	128	32	128	1	2	2	0	4	0	0	0	0	0	0	0	0	0	0	0	0	0
5142	HCC8	*C. jejuni*	128	32	64	1	2	1	15	4	0	0	0	0	0	0	0	0	0	0	0	0	0
5143	HCC9	*C. jejuni*	64	8	64	0.125	2	0.5	0	29	0	0	0	0	0	0	0	0	0	0	0	0	0
5144	HCC10	*C. jejuni*	256	32	2	2	2	2	0	0	0	0	0	0	0	0	0	0	0	0	0	0	0
5145	HCC11	*C. jejuni*	256	32	128	1	4	2	12	30	0	0	0	0	0	0	0	0	0	0	0	0	0
5146	HCC13	*C. jejuni*	256	32	128	1	4	2	0	31	0	0	0	0	0	0	0	0	0	0	0	0	0
5147	HCC16	*C. jejuni*	256	32	4	1	2	2	5	0	0	0	0	0	0	0	0	0	0	0	0	0	0
5148	HCC17	*C. jejuni*	8	0.25	128	1	2	2	16	4	0	0	0	0	0	0	0	0	0	0	0	0	0
5149	HCC18	*C. jejuni*		64	4	1	4	2	5	0	0	0	0	0	0	0	0	0	0	0	0	0	0
5150	HCC19	*C. jejuni*	256	32	128	1	2	1	5	17	0	0	0	0	0	0	0	0	0	0	0	0	0
5151	HCC20	*C. jejuni*	256	512	>512	2	4	2	12	32	0	0	0	0	0	0	0	0	0	0	0	0	0
5152	HCC21	*C. jejuni*	256	32	64	1	4	2	17	28	0	0	0	0	0	0	0	0	0	0	0	0	0
5153	HCC22	*C. jejuni*	256	32	128	1	4	2	5	33	0	0	0	0	0	0	0	0	0	0	0	0	0
5154	HCC23	*C. jejuni*	512	512	>512	2	4	2	5	18	0	0	0	0	0	0	0	0	0	0	0	0	0
5155	HCC24	*C. jejuni*	256	32	128	1	2	2	18	4	0	0	0	0	0	0	0	0	0	0	0	0	0
5156	HCC25	*C. jejuni*	256	32	128	1	4	2	0	17	0	0	0	0	0	0	0	0	0	0	0	0	0
5157	HCC26	*C. jejuni*	8	1	1	2	8	2	5	0	0	0	0	0	0	0	0	0	0	0	0	0	0
5158	HCC27	*C. jejuni*		1	2	1	8	4	5	0	0	0	0	0	0	0	0	0	0	0	0	0	0
5159	HCC29	*C. jejuni*	256	32	256	1	4	2	5	12	6	11	0	0	0	0	0	0	0	0	0	0	0
5160	HCC30	*C. jejuni*	128	32	256	1	8	2	0	0	0	0	0	0	0	0	0	0	0	0	0	0	0
5161	HCC31	*C. jejuni*	8	0.5	4	1	4	2	12	0	0	0	0	0	0	0	0	0	0	0	0	0	0
5162	HCC32	*C. jejuni*	256	128	8	2	8	2	0	0	0	0	0	0	0	0	0	0	0	0	0	0	0
5163	HCC33	*C. jejuni*	256	32	64	2	4	2	5	33	0	0	0	0	0	0	0	0	0	0	0	0	0
5164	HCC34	*C. jejuni*	32	2	4	1	8	2	0	0	0	0	0	0	0	0	0	0	0	0	0	0	0
5165	HCC35	*C. jejuni*	8	0.25	1	1	2	2	12	0	0	0	0	0	0	0	0	0	0	0	0	0	0
5166	HCC36	*C. jejuni*	256	64	4	2	4	2	19	0	0	0	0	0	0	0	0	0	0	0	0	0	0
5167	HCC37	*C. jejuni*	128	32	128	2	2	2	0	12	0	0	0	0	0	0	0	0	0	0	0	0	0
5168	HCC38	*C. jejuni*	64	16	64	0.5	2	1	0	29	0	0	0	0	0	0	0	0	0	0	0	0	0
5169	HCC40	*C. jejuni*	128	64	64	2	8	1	5	34	0	0	0	0	0	0	0	0	0	0	0	0	0
5170	HCC41	*C. coli*	128	64	64	2	4	2	0	4	0	0	0	0	0	0	0	0	0	0	0	0	0
5171	HCC43	*C. coli*	128	128	512	8	4	2	0	4	0	0	0	0	0	0	0	0	0	0	0	0	0
5172	HCC44	*C. jejuni*	128	64	0.5	2	2	2	20	0	0	0	0	0	0	0	0	0	0	0	0	0	0
5173	HCC45	*C. jejuni*	128	64	128	1	4	2	5	17	0	0	0	0	0	0	0	0	0	0	0	0	0
5174	HCC46	*C. jejuni*	256	64	16	2	2	1	5	28	0	0	0	0	0	0	0	0	0	0	0	0	0
5175	HCC47	*C. jejuni*	256	64	128	1	2	2	5	4	0	0	0	0	0	0	0	0	0	0	0	0	0
5176	HCC48	*C. jejuni*	256	64	512	1	4	2	0	17	0	0	0	0	0	0	0	0	0	0	0	0	0
5177	HCC49	*C. jejuni*	256	64	128	1	4	2	5	4	0	0	0	0	0	0	0	0	0	0	0	0	0
5178	HCC50	*C. jejuni*	64	64	512	2	2	2	21	35	0	0	0	0	0	0	0	0	0	0	0	0	0
5180	HCC52	*C. jejuni*	128	32	128	0.5	2	2	0	29	0	0	0	0	0	0	0	0	0	0	0	0	0

5181	HCC54	*C. jejuni*	8	1	128	2	4	2	22	36	0	0	0	0	0	0	0	0	0	0	0	0	0
5182	HCC55	*C. jejuni*	256	32	512	1	2	2	0	17	0	0	0	0	0	0	0	0	0	0	0	0	0
5183	HCC57	*C. jejuni*	256	64	2	1	4	2	0	0	0	0	0	0	0	0	0	0	0	0	0	0	0
5184	HCC58	*C. jejuni*	256	64	128	2	4	2	0	17	0	0	0	0	0	0	0	0	0	0	0	0	0
5185	HCC59	*C. jejuni*	256	64	512	2	2	2	0	4	0	0	0	0	0	0	0	0	0	0	0	0	0
5186	HCC60	*C. jejuni*	256	64	512	2	2	2	12	35	0	0	0	0	0	0	0	0	0	0	0	0	0
5187	HCC61	*C. jejuni*	256	64	16	1	1	2	5	28	0	0	0	0	0	0	0	0	0	0	0	0	0
5188	HCC62	*C. jejuni*	256	64	4	0.5	4	2	23	0	0	0	0	0	0	0	0	0	0	0	0	0	0
5189	HCC63	*C. jejuni*	256	512	512	4	4	2	0	17	0	0	0	0	0	0	0	0	0	0	0	0	0
5190	HCC64	*C. jejuni*		1	128	1	2	2	16	4	0	0	0	0	0	0	0	0	0	0	0	0	0
5191	HCC65	*C. jejuni*		1	256	1	1	1	0	37	0	0	0	0	0	0	0	0	0	0	0	0	0
5192	HCC66	*C. jejuni*		128	32	2	2	2	24	0	0	0	0	0	0	0	0	0	0	0	0	0	0
5193	HCC67	*C. jejuni*		0.25	256	1	2	1	0	0	0	0	0	0	0	0	0	0	0	0	0	0	0
5194	HCC68	*C. jejuni*		32	128	1	2	1	0	28	0	0	0	0	0	0	0	0	0	0	0	0	0
5195	HCC69	*C. jejuni*		64	4	2	4	2	25	0	0	0	0	0	0	0	0	0	0	0	0	0	0
5196	HCC70	*C. jejuni*		32	64	2	2	1	0	28	0	0	0	0	0	0	0	0	0	0	0	0	0
5197	HCC71	*C. jejuni*		32	64	1	2	2	12	17	0	0	0	0	0	0	0	0	0	0	0	0	0
5198	HCC72	*C. jejuni*		16	128	2	2	2	5	12	0	0	0	0	0	0	0	0	0	0	0	0	0
5199	HCC73	*C. jejuni*		32	64	1	2	2	8	17	0	0	0	0	0	0	0	0	0	0	0	0	0
5200	HCC74	*C. jejuni*		32	64	2	2	2	0	31	0	0	0	0	0	0	0	0	0	0	0	0	0
5201	HCC75	*C. jejuni*		32	128	2	4	2	5	12	0	0	0	0	0	0	0	0	0	0	0	0	0
5202	HCC76	*C. jejuni*		32	128	1	4	1	5	12	0	0	0	0	0	0	0	0	0	0	0	0	0
5203	HCC77	*C. jejuni*		64	512	2	2	2	0	12	0	0	0	0	0	0	0	0	0	0	0	0	0
5204	HCC79	*C. jejuni*		32	2	1	2	2	0	0	0	0	0	0	0	0	0	0	0	0	0	0	0
5205	HCC83	*C. jejuni*		16	128	1	2	1	12	4	0	0	0	0	0	0	0	0	0	0	0	0	0
5206	HCC84	*C. jejuni*		32	64	1	2	2	5	0	0	0	0	0	0	0	0	0	0	0	0	0	0
5207	HCC86	*C. coli*		128	128	2	128	2	1	16	0	3	0	0	0	0	0	0	0	0	0	0	0
5208	HCC87	*C. jejuni*		32	2	0.5	4	2	25	0	0	0	0	0	0	0	0	0	0	0	0	0	0
5209	HCC88	*C. jejuni*		32	64	1	2	2	10	29	0	0	0	0	0	0	0	0	0	0	0	0	0
5210	HCC89	*C. jejuni*		32	64	1	2	2	8	17	0	0	0	0	0	0	0	0	0	0	0	0	0
5211	HCC90	*C. jejuni*		32	128	2	4	2	10	29	0	0	0	0	0	0	0	0	0	0	0	0	0
5212	HCC91	*C. jejuni*		16	64	1	2	2	12	18	0	0	0	0	0	0	0	0	0	0	0	0	0
5213	HCC92	*C. jejuni*		32	64	1	2	2	0	17	0	0	0	0	0	0	0	0	0	0	0	0	0
5215	HCC94	*C. jejuni*	NT	NT	NT	NT	NT	NT	0	17	0	0	0	0	0	0	0	0	0	0	0	0	0
5216	HCC95	*C. jejuni*		16	64	0.5	2	2	8	17	0	0	0	0	0	0	0	0	0	0	0	0	0
5217	HSA1	*C. jejuni*	64	16	64	1	4	2	5	29	0	0	0	0	0	0	0	0	0	0	0	0	0
5218	HSA2	*C. jejuni*	128	16	128	1	2	2	5	29	0	0	0	0	0	0	0	0	0	0	0	0	0
5219	HSA3	*C. jejuni*	256	16	64	1	4	1	0	0	0	0	0	0	0	0	0	0	0	0	0	0	0
5220	HSA4	*C. jejuni*	256	16	64	2	4	1	0	0	0	0	0	0	0	0	0	0	0	0	0	0	0
5221	HSA5	*C. jejuni*	128	64	256	1	2	2	26	12	0	0	0	0	0	0	0	0	0	0	0	0	0
5222	HSA6	*C. jejuni*	128	16	64	1	4	1	27	4	0	0	0	0	0	0	0	0	0	0	0	0	0
5223	HSA7	*C. jejuni*	128	16	4	2	1	1	0	0	0	0	0	0	0	0	0	0	0	0	0	0	0
5224	HSA8	*C. jejuni*	128	32	64	1	4	2	5	33	0	0	0	0	0	0	0	0	0	0	0	0	0
5225	HSA9	*C. jejuni*	64	16	64	0.5	2	1	28	17	0	0	0	0	0	0	0	0	0	0	0	0	0
5226	HSA10	*C. jejuni*	128	16	128	0.5	2	2	16	12	0	0	0	0	0	0	0	0	0	0	0	0	0
5227	HSA11	*C. jejuni*	128	32	128	1	4	1	5	38	0	0	0	0	0	0	0	0	0	0	0	0	0
5228	HSA12	*C. jejuni*	4	0.125	1	0.5	4	2	5	0	0	0	0	0	0	0	0	0	0	0	0	0	0
5229	HSA13	*C. jejuni*	128	32	128	0,5/1	4	2	26	17	0	0	0	0	0	0	0	0	0	0	0	0	0
5230	HSA14	*C. jejuni*	8	0.25	4	1	4	1	25	0	0	0	0	0	0	0	0	0	0	0	0	0	0
5231	HSA16	*C. coli*	16	16	32	1	8	1	0	4	0	0	0	0	0	0	0	0	0	0	0	0	0
5233	HSA17	*C. jejuni*	128	8	32	1	4	2	0	39	0	0	0	0	0	0	0	0	0	0	0	0	0
5234	HSA18	*C. jejuni*		0.25	1	0.5	4	2	23	0	0	0	0	0	0	0	0	0	0	0	0	0	0
5235	HSA19	*C. jejuni*	8	0.25	1	0.5	4	2	0	0	0	0	0	0	0	0	0	0	0	0	0	0	0
5236	HSA20	*C. coli*	32	16	64	1	4	1	0	4	0	0	0	0	0	0	0	0	0	0	0	0	0
5237	HSA21	*C. jejuni*	256	32	128	2	4	2	26	17	0	0	0	0	0	0	0	0	0	0	0	0	0
5238	HSA22	*C. jejuni*	128	16	128	1	4	2	0	40	0	0	0	0	0	0	0	0	0	0	0	0	0
5239	HSA23	*C. jejuni*	128	64	256	8	2	1	0	41	0	0	0	0	0	0	0	0	0	0	0	0	0
5240	HSA24	*C. jejuni*	128	16	128	2	4	1	5	4	0	0	0	0	0	0	0	0	0	0	0	0	0
5242	HSA26	*C. jejuni*	256	64	64	1	2	2	0	4	0	0	0	0	0	0	0	0	0	0	0	0	0
5243	HSA27	*C. jejuni*	128	32	128	1	4	2	26	17	0	0	0	0	0	0	0	0	0	0	0	0	0
5245	HSA29	*C. jejuni*	128	32	128	4	2	1	5	41	0	0	0	0	0	0	0	0	0	0	0	0	0
5246	HSA30	*C. jejuni*	8	1	128	2	4	0.5	22	36	0	0	0	0	0	0	0	0	0	0	0	0	0
5247	HSA31	*C. jejuni*	128	16	1	2	4	1	0	0	0	0	0	0	0	0	0	0	0	0	0	0	0
5248	HSA32	*C. jejuni*	128	32	128	2	>512	2	16	12	0	0	0	0	0	0	0	0	0	0	0	0	0
5249	HSA33	*C. jejuni*	256	32	64	4	2	2	29	28	0	0	0	0	0	0	0	0	0	0	0	0	0
5250	HSA35	*C. jejuni*	128	16	128	1	4	2	28	17	0	0	0	0	0	0	0	0	0	0	0	0	0
5251	HSA36	*C. jejuni*	128	32	64	2	2/1	1	0	4	0	0	0	0	0	0	0	0	0	0	0	0	0
5252	HSA37	*C. jejuni*	128	16	128	1	4	2	26	17	0	0	0	0	0	0	0	0	0	0	0	0	0
5253	HSA38	*C. jejuni*	128	64	>512	2	4	2	0	4	0	0	0	0	0	0	0	0	0	0	0	0	0
5254	HSA39	*C. jejuni*	256	64	16	2	2	2	0	28	0	0	0	0	0	0	0	0	0	0	0	0	0
5255	HSA40	*C. jejuni*	128	32	128	1	>512	2	16	12	0	0	0	0	0	0	0	0	0	0	0	0	0
5256	HSA41	*C. jejuni*	128	16	4	1	2	2	0	0	0	0	0	0	0	0	0	0	0	0	0	0	0
5257	HSA42	*C. jejuni*	128	16	128	2	2	1	0	17	0	0	0	0	0	0	0	0	0	0	0	0	0
5258	HSA43	*C. jejuni*	128	16	128	1	2	2	15	4	0	0	0	0	0	0	0	0	0	0	0	0	0
5259	HSA44	*C. jejuni*	128	32	64	2	2	2	5	33	0	0	0	0	0	0	0	0	0	0	0	0	0
5260	HSA45	*C. jejuni*	128	32	128	1	2	1	0	29	0	0	0	0	0	0	0	0	0	0	0	0	0
5261	HSA46	*C. coli*	8	0.25	256	2	64	2	0	4	0	5	0	0	0	0	0	0	0	0	0	0	0
5262	HSA47	*C. jejuni*	8	0.5/1	4	1	2	1	0	0	0	0	0	0	0	0	0	0	0	0	0	0	0
5263	HSA48	*C. jejuni*	128	16	128	2	2	1	5	17	0	0	0	0	0	0	0	0	0	0	0	0	0
5264	AV.01	*C. jejuni*	128	8	32	0.5	1	0.5	10	12	0	0	0	0	0	0	0	0	0	0	0	0	0
5267	AV.04	*C. jejuni*	128	8	32	64	1	1	12	4	0	0	0	0	0	0	0	0	0	0	0	0	0
5270	AV.07	*C. jejuni*	128	8	32	64	1	1	5	42	0	0	0	0	0	0	0	0	0	0	0	0	0
5271	AV.08	*C. jejuni*	4	0.06	32	64	1	0.5	0	28	0	0	0	0	0	0	0	0	0	0	0	0	0
5272	AV.09	*C. jejuni*	128	8	32	0.5	32	1	30	17	0	0	10	5	0	0	0	2	0	0	0	0	0
5274	AV.11	*C. jejuni*	128	8	32	0.5	32	1	31	43	0	0	8	6	0	0	0	1	0	0	0	0	0
5275	AV.12	*C. jejuni*	128	8	32	0.5	32	1	0	0	0	0	9	4	0	2	0	0	0	0	0	0	0
5276	AV.13	*C. jejuni*	128	8	32	0.5	32	1	5	44	0	0	0	0	0	2	0	0	0	0	0	1	0
5279	AV.16	*C. jejuni*	128	8	32	0.5	1	2	0	30	0	0	0	0	0	0	0	0	0	0	0	0	0
5281	AV.18	*C. jejuni*	128	8	0.25	0.5	4	2	32	0	0	0	0	0	0	0	0	0	0	0	0	0	0
5282	AV.19	*C. jejuni*	128	8	32	2	1	1	5	19	0	0	0	0	0	0	0	0	0	0	0	0	0
5284	AV.21	*C. jejuni*	128	8	32	0.5	1	1	16	12	0	0	0	0	0	0	0	0	0	0	0	0	0
5285	AV.22	*C. jejuni*	128	8	32	0.5	32	2	5	44	0	0	0	0	0	2	0	0	0	0	0	1	0
5286	AV.23	*C. jejuni*	128	8	32	0.5	32	2	33	12	0	0	0	0	0	0	0	0	0	0	0	0	0
5287	AV.24	*C. jejuni*	128	8	32	0.5	32	1	5	44	0	0	0	0	0	2	0	0	0	0	0	1	0
5288	AV.25	*C. jejuni*	128	8	0.25	0.5	4	2	0	0	0	0	0	0	0	0	0	0	0	0	0	0	0
5289	AV.26	*C. jejuni*	128	8	32	0.5	1	2	0	33	0	0	0	0	0	0	0	0	0	0	0	0	0
5291	AV.28	*C. jejuni*	128	8	32	0.5	1	0.5	34	4	0	0	0	0	0	0	0	0	0	0	0	0	0
5293	BO.02	*C. jejuni*	128	8	32	0.5	4	1	0	17	0	0	0	0	0	0	0	0	0	0	0	0	0
5295	BO.04	*C. jejuni*	1	0.12	16	0.5	16	2	5	41	0	0	0	0	0	0	0	0	0	0	0	0	0
5296	BO.05	*C. jejuni*	128	8	32	0.5	4	1	5	0	0	0	0	0	0	0	0	0	0	0	0	0	0
5297	BO.06	*C. jejuni*	128	8	32	0.5	1	1	15	4	0	0	0	0	0	0	0	0	0	0	0	0	0
5298	BO.07	*C. jejuni*	8	0.12	32	0.5	1	1	0	17	0	0	0	0	0	0	0	0	0	0	0	0	0
5299	BO.08	*C. jejuni*	8	0.12	32	0.5	1	1	5	28	0	0	0	0	0	0	0	0	0	0	0	0	0
5300	BO.09	*C. jejuni*	128	8	32	1	32	2	5	41	0	0	0	0	0	0	0	0	0	0	0	0	0
5301	BO.10	*C. jejuni*	128	8	32	2	8	2	5	36	0	0	0	0	0	0	0	0	0	0	0	0	0
5302	BO.11	*C. jejuni*	8	0.12	0.25	0.5	4	2	0	0	0	0	0	0	0	0	0	0	0	0	0	0	0
5303	BO.12	*C. jejuni*	8	0.12	32	0.5	1	0.5	5	0	0	0	0	0	0	0	0	0	0	0	0	0	0
5304	BO.13	*C. jejuni*	128	8	32	1	32	2	0	0	0	0	0	0	0	0	0	0	0	0	0	0	0
5305	BO.14	*C. jejuni*	128	8	32	1	32	2	5	41	0	0	0	0	0	0	0	0	0	0	0	0	0
5306	BO.15	*C. jejuni*	128	8	32	0.5	4	1	0	12	7	12	0	0	0	0	0	0	0	0	0	0	0
5307	BO.16	*C. jejuni*	128	8	32	0.5	1	1	5	45	0	0	0	0	0	0	0	0	0	0	0	0	0
5308	BO.17	*C. jejuni*	1	8	32	0.5	128	2	5	17	0	0	0	0	0	0	0	0	0	0	0	0	0
5309	BO.18	*C. jejuni*	1	0.12	0.25	0.5	4	2	8	0	0	0	0	0	0	0	0	0	0	0	0	0	0
5310	BO.19	*C. coli*	128	8	32	1	32	2	1	20	2	6	0	0	0	0	0	0	0	0	0	0	0
5311	BO.20	*C. jejuni*	128	8	32	1	32	1	5	0	0	0	9	4	0	2	0	0	0	0	0	0	0
5313	BO.22	*C. jejuni*	128	8	32	0.5	2	1	5	46	0	0	0	0	0	0	0	0	0	0	0	0	0
5316	BO.25	*C. jejuni*	128	8	32	0.5	1	0.5	0	46	0	0	0	0	0	0	0	0	0	0	0	0	0
5317	BO.26	*C. jejuni*	8	0.06	32	0.5	1	1	5	28	0	0	0	0	0	0	0	0	0	0	0	0	0
5318	BO.27	*C. jejuni*	64	8	32	0.5	1	0.5	5	46	0	0	0	0	0	0	0	0	0	0	0	0	0
5321	BO.30	*C. jejuni*	128	8	32	0.5	1	1	5	47	0	0	0	0	0	0	0	0	0	0	0	0	0
5322	BO.31	*C. jejuni*	32	8	32	0.5	1	0.25	5	48	0	0	11	4	0	0	0	1	0	0	0	0	0
5324	BO.33	*C. jejuni*	128	8	32	2	32	1	32	29	0	0	0	0	0	0	0	0	0	0	0	0	0
5325	BO.34	*C. jejuni*	64	8	0.25	0.5	1	1	35	0	0	0	0	0	0	0	0	0	0	0	0	0	0
5327	BO.36	*C. jejuni*	64	8	32	0.5	1	0.5	0	41	0	0	0	0	0	0	0	0	0	0	0	0	0
