# Supplementary figures and images for: Gene pool transmission of multidrug resistance among Campylobacter from livestock, sewage and human disease
Source: Environ Microbiol. 2019 Aug 27;21(12):4597–613. doi: 10.1111/1462-2920.14760 (PMC6916351; doi:10.1111/1462-2920.14760)

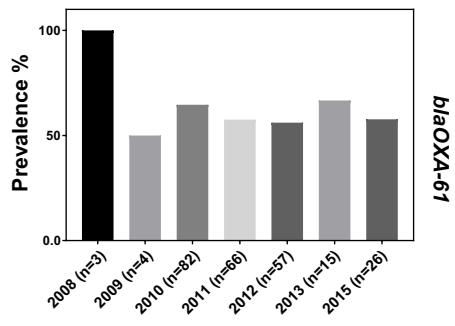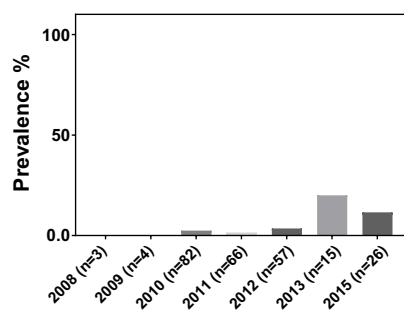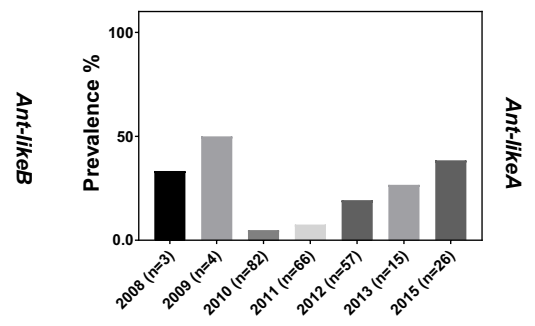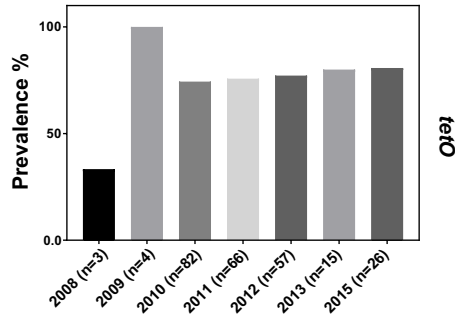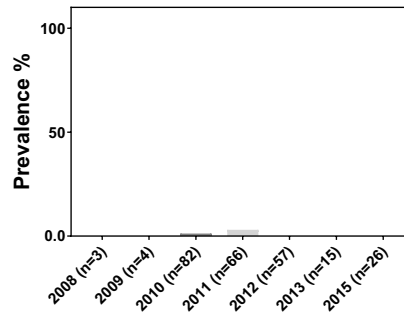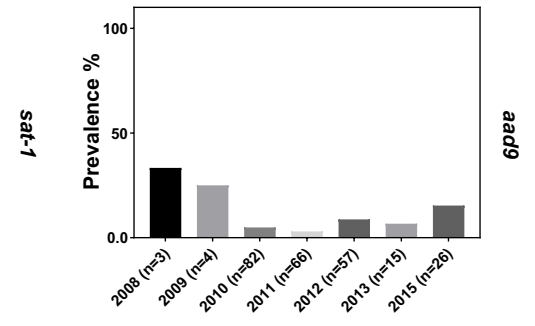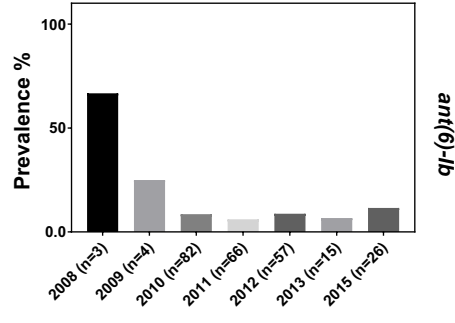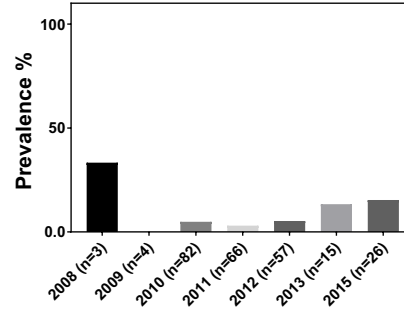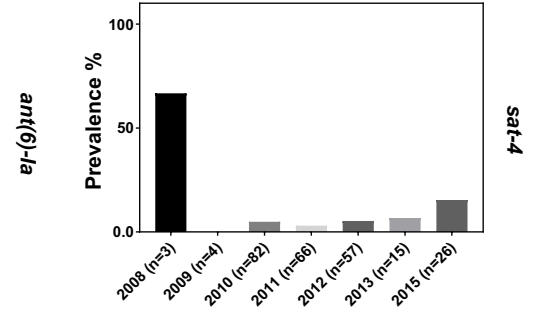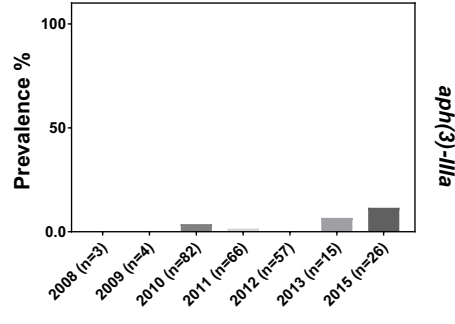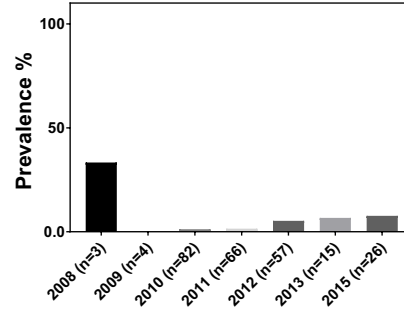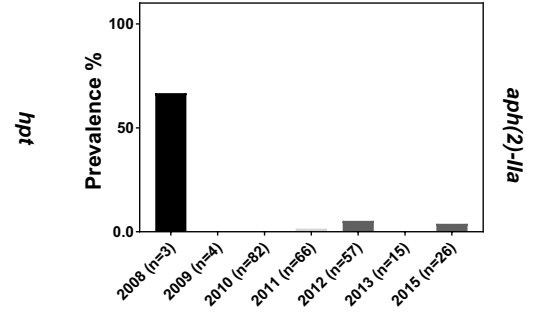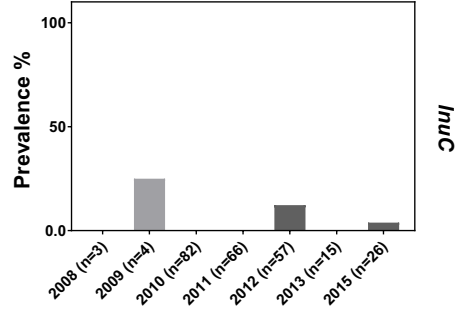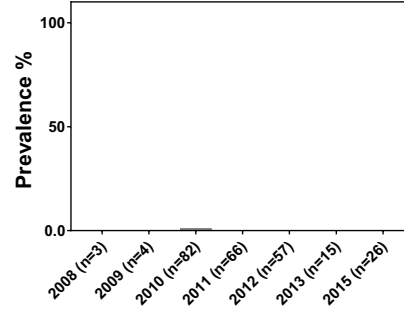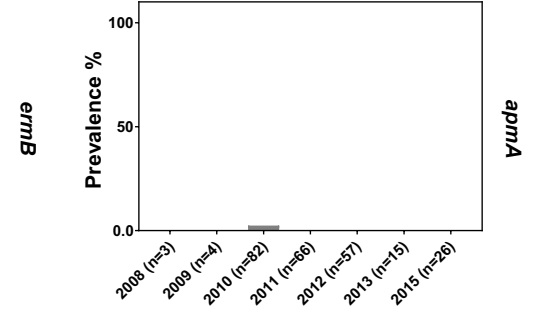

Supplement: Supplementary file 7 — Fig. S2. Prevalence of AMR genes over time. Graphs illustrate the presence of 15 putative AMR genes in isolate genomes sampled at each year in the study. Prevalence (%) was calculated by dividing the number of samples that had the AMR gene by the total number of samples in that year. [file EMI-21-4597-s002.pdf]
